# Caveolin-1 mediates blood-brain barrier permeability, neuroinflammation, and cognitive impairment in SARS-CoV-2 infection

**DOI:** 10.1016/j.jneuroim.2024.578309

**Published:** 2024-02-04

**Authors:** Troy N. Trevino, Ali A. Almousawi, KaReisha F. Robinson, Avital B. Fogel, Jake Class, Richard D. Minshall, Leon M. Tai, Justin M. Richner, Sarah E. Lutz

**Affiliations:** a Departments of Anatomy and Cell Biology, University of Illinois at Chicago College of Medicine, USA; b Departments of Microbiology and Immunology, University of Illinois at Chicago College of Medicine, USA; c Departments of Anesthesiology, and Pharmacology and Regenerative Medicine, University of Illinois at Chicago College of Medicine, USA

**Keywords:** SARS-CoV-2, Caveolin-1, Claudin-5, Blood-brain barrier, CD3, T cell, Neuroinflammation, Novel object recognition, Endothelial, Brain

## Abstract

Blood-brain barrier (BBB) permeability can cause neuroinflammation and cognitive impairment. Caveolin-1 (Cav-1) critically regulates BBB permeability, but its influence on the BBB and consequent neurological outcomes in respiratory viral infections is unknown. We used Cav-1-deficient mice with genetically encoded fluorescent endothelial tight junctions to determine how Cav-1 influences BBB permeability, neuroinflammation, and cognitive impairment following respiratory infection with mouse adapted (MA10) SARS-CoV-2 as a model for COVID-19. We found that SARS-CoV-2 infection increased brain endothelial Cav-1 and increased transcellular BBB permeability to albumin, decreased paracellular BBB Claudin-5 tight junctions, and caused T lymphocyte infiltration in the hippocampus, a region important for learning and memory. Concordantly, we observed learning and memory deficits in SARS-CoV-2 infected mice. Importantly, genetic deficiency in Cav-1 attenuated transcellular BBB permeability and paracellular BBB tight junction losses, T lymphocyte infiltration, and gliosis induced by SARS-CoV-2 infection. Moreover, Cav-1 KO mice were protected from the learning and memory deficits caused by SARS-CoV-2 infection. These results establish the contribution of Cav-1 to BBB permeability and behavioral dysfunction induced by SARS-CoV-2 neuroinflammation.

## 1. Introduction

Brain endothelial cells regulate the permeability of the cere-brovasculature, referred to as the blood brain barrier (BBB). BBB destabilization causes extravasation of blood proteins and immune cells into the CNS, leading to neuroinflammation, pruning of synapses and neurons, and behavioral changes (Merlini et al., 2019). BBB permeability in the hippocampus particularly contributes to cognitive impairment, consistent with the important role of the hippocampus in cognition (Garber et al., 2019). Disorders of cognition are frequent in COVID-19 (Spudich and Nath, 2022), and BBB permeability and neuroinflammation are pronounced in COVID-19 decedents and animal models (Fernández-Castañeda et al., 2022; Krasemann et al., 2022; Lee et al., 2021; Lee et al., 2022; Matschke et al., 2020; Soung et al., 2022; Thakur et al., 2021; Zhang et al., 2021). Indeed, the hippocampus is a target of inflammation and neurodegeneration in COVID-19 patients (Bayat et al., 2022; Nouraeinejad, 2023; Soung et al., 2022); hippocampal atrophy correlates to cognitive decline after COVID-19 (Douaud et al., 2022). Emerging evidence links infiltration of blood proteins and T cells into the CNS to neuroinflammatory processes in COVID-19 (Heming et al., 2021; Lee et al., 2021; Schwabenland et al., 2021; Soung et al., 2022; Vanderheiden and Klein, 2022). However, the contribution of BBB permeability to neuroinflammation and cognitive impairment in COVID-19 is incompletely understood. We designed the present study to address how the effect of SARS-CoV-2 infection on specific aspects of the BBB would influence cognition.

Here, we focused on Caveolin-1 (Cav-1) (Jones and Minshall, 2020; Ohi and Kenworthy, 2022; Parton, 2018). Cav-1 is involved in multiple aspects of BBB permeability. Cav-1 contributes to endocytosis and transcellular transcytosis of macromolecules and cells from the blood to the brain (Andreone et al., 2017; Liebner et al., 2018; Pandit et al., 2020; Pol et al., 2020). Indeed, gradual suppression of Cav-1 during development is required for the formation of the transcellular BBB (Chow and Gu, 2017), whereas Cav-1 upregulation reopens the transcellular BBB in the adult (Ayloo and Gu, 2019; Chang et al., 2022; Lutz et al., 2017; Pandit et al., 2020). Second, Cav-1 can modify paracellular permeability *via* endocytosis and removal of tight junctions from paracellular junctions (Li et al., 2015; Liu et al., 2012; Marchiando et al., 2010; Nag et al., 2007; Song et al., 2007; Stamatovic et al., 2009). Third, Cav-1 influences the scaffolding and membrane distribution of leukocyte adhesion molecules important for leukocyte transendothelial migration, including vascular cell adhesion molecule (VCAM)-1 and intracellular adhesion molecule (ICAM)-1 (Carman and Springer, 2004; Dragoni et al., 2017; Han et al., 2010; Heemskerk et al., 2016; Hu et al., 2008; Kanters et al., 2008; Lolo et al., 2022; Millan et al., 2006; Wu et al., 2016; Xu et al., 2013). Indeed, we have shown that Cav-1 promotes extravasation of albumin, pro-encephalitogenic T lymphocytes, and neutrophils across the endothelium (Hu et al., 2008; Lutz et al., 2017; Tiruppathi et al., 2004). Thus, changes in endothelial endocytosis, transcytosis, and altered protein distribution due to upregulation of Cav-1 are poised to influence multiple aspects of endothelial cell function in disease. In fact, cerebrovascular Cav-1 protein and activity is increased coincident with or prior to BBB opening and leukocyte infiltration in mouse models for stroke (Knowland et al., 2014), multiple sclerosis (Errede et al., 2012; Kim et al., 2006; Wu et al., 2016), and traumatic injury (Nag et al., 2007; Zhang et al., 2022). Furthermore, suppressing Cav-1 reduces leukocyte adhesion, BBB migration, neuroinflammation, and neurodegeneration *in vivo* (Lutz et al., 2017; Wu et al., 2016; Zhang et al., 2022). Strikingly, Cav-1 is upregulated in forebrains of COVID-19 decedents (Green et al., 2022; Premkumar and Sajitha, 2023). However, the extent to which Cav-1 contributes to BBB permeability, neuroinflammation, and cognitive impairment in COVID-19 has not been tested.

Thus, the goal of the present study was to evaluate the extent to which Cav-1 contributes to cognitive impairment by promoting BBB permeability in a COVID-19 mouse model. For this, we deployed a mouse adapted strain of SARS-CoV-2 (MA10) that has been generated by engineering the spike protein to bind to the murine homolog of the viral entry receptor, ACE2, and sequential passages of the mouse-adapted strain through mice (Leist et al., 2020). Intranasal inoculation with SARS-CoV-2 MA10 infects ACE2-expressing alveolar epithelium and alveolar type II (AT2) cells within the lung, yielding acute respiratory infection (Leist et al., 2020). Recently, MA10 has emerged as a promising tool to study mechanisms of SARS-CoV-2 neuropathogenesis because it recapitulates features of COVID-19 neuroinflammation, with greater severity in the aged (Amruta et al., 2022). We found that respiratory infection with SARS-CoV-2 MA10 upregulated Cav-1 and decreased Claudin-5 in brain endothelial cells. This was accompanied by heightened permeability to intravenously injected albumin, T cell infiltration, neuroinflammation, and learning/memory deficits in infected mice. Importantly, genetic deficiency in Cav-1 offered protection from SARS-CoV-2 induced BBB permeability, neuroinflammation and memory deficits. These data indicate that Cav-1-mediated BBB permeability is increased during acute SARS-CoV-2 infection and may contribute to neuropathology and cognitive impairment in COVID-19.

## 2. Results

### 2.1. SARS-CoV-2 infection increases cerebrovascular Cav-1

To induce the mouse adapted SARS-CoV-2 MA10 model, we infected 12-month-old C57Bl/6 Cav-1^+/+^ (WT) mice and 12-month old Cav-1^−/−^ mice with SARS-CoV-2 (strain MA10) by intranasal inoculation. The MA10 model has been previously characterized (Amruta et al., 2022; Leist et al., 2020). 12-month-old mice were used to recapitulate the age-related morbidity of SARS-CoV-2 infection (Amruta et al., 2022; Bartleson et al., 2021; Davis et al., 2022; O’Driscoll et al., 2021; Oishi et al., 2022; Ostendorf et al., 2022; Sullivan and Fischer, 2021). We found that mice infected with MA10 exhibited <10% body weight loss and transient decrease in arteriole oxygenation (Supplementary Fig. 2 A–D). At euthanasia at 5 days post inoculation (5 DPI), viral RNA in the lung was detected at ~10^7^ viral genomes/mg tissue (Supplementary Fig. 2E). Cav-1^−/−^ mice exhibited a non-significant trend toward greater body weight prior to inoculation (Supplementary Fig. 2 A–B). Cav-1 genotype did not change quantity of RNA detected in the lung, weight loss, or arterial oxygenation in SARS-CoV-2 infection (Supplementary Fig. 2 A–E).

**Fig. 1.**
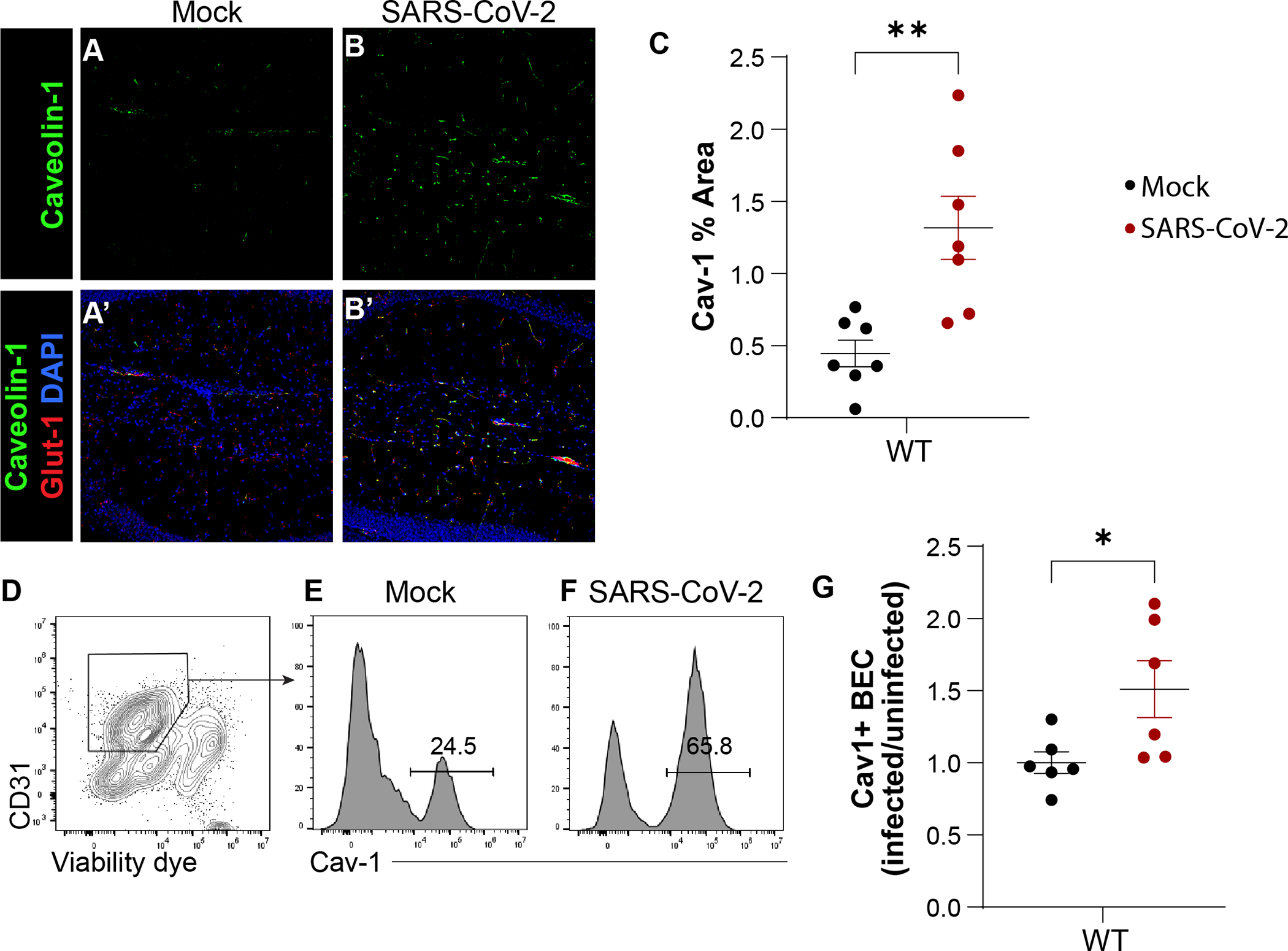
Cav-1 is increased in brain endothelial cells in SARS-CoV-2 infection. A-B) Immunofluorescence detection of Cav-1 (green) in hippocampal sections from wild-type mice euthanized 4 days post inoculation (DPI) with SARS-CoV-2. Monochromatic images (A, B) and overlays between Caveolin-1 (green), brain endothelial cell protein Glut-1 (red), and DAPI (blue) (A’, B′). C) Quantification of % area immunoreactive for Cav-1 in hippocampus sections. Three brain sections analyzed per mouse. **p < 0.01, unpaired student *t*-test. D) Flow cytometric plot of CD31 and viable exclusion dye demonstrates gating strategy for endothelial cells (box) in single cell suspension prepared from isolated brain microvessels of WT mice euthanized 4 days after respiratory inoculation with SARS-CoV-2. *E*-F) Histograms of Cav-1 fluorescence intensity in viable CD31+ endothelial cells isolated as in D. G) Quantification of Cav-1 fluorescence intensity in viable CD31+ endothelial cells from mice euthanized at 4 DPI with SARS-CoV-2, expressed as ratio to healthy WT. *n* = 6 mice/group from two independent experiments. *p* < 0.05, unpaired student t-test.

We next tested if Cav-1 is altered at the BBB during SARS-CoV-2 infection. At 4 DPI, mice were euthanized and Cav-1 expression in brain microvessels was assessed by immunostaining (Fig. 1A–C). We found that SARS-CoV-2 infection significantly increased Cav-1 positive area in the hippocampus, with a vascular distribution (Fig. 1C). Similarly, Cav-1 expression was high in the olfactory bulb, cortex, and brainstem in SARS-CoV-2 infected mice (Supplementary Fig. 3). We confirmed these results by quantifying Cav-1 by flow cytometry on endothelial cells acutely isolated from whole mouse brain. The percentage of brain endothelial cells with high Cav-1 expression was increased by SARS-CoV-2 infection (Fig. 1D–G). These data indicate that infection with SARS-CoV-2 upregulates Cav-1 in brain endothelial cells.

### 2.2. Cav-1 promotes transcellular and paracellular BBB permeability in SARS-CoV-2 infection

**Fig. 2.**
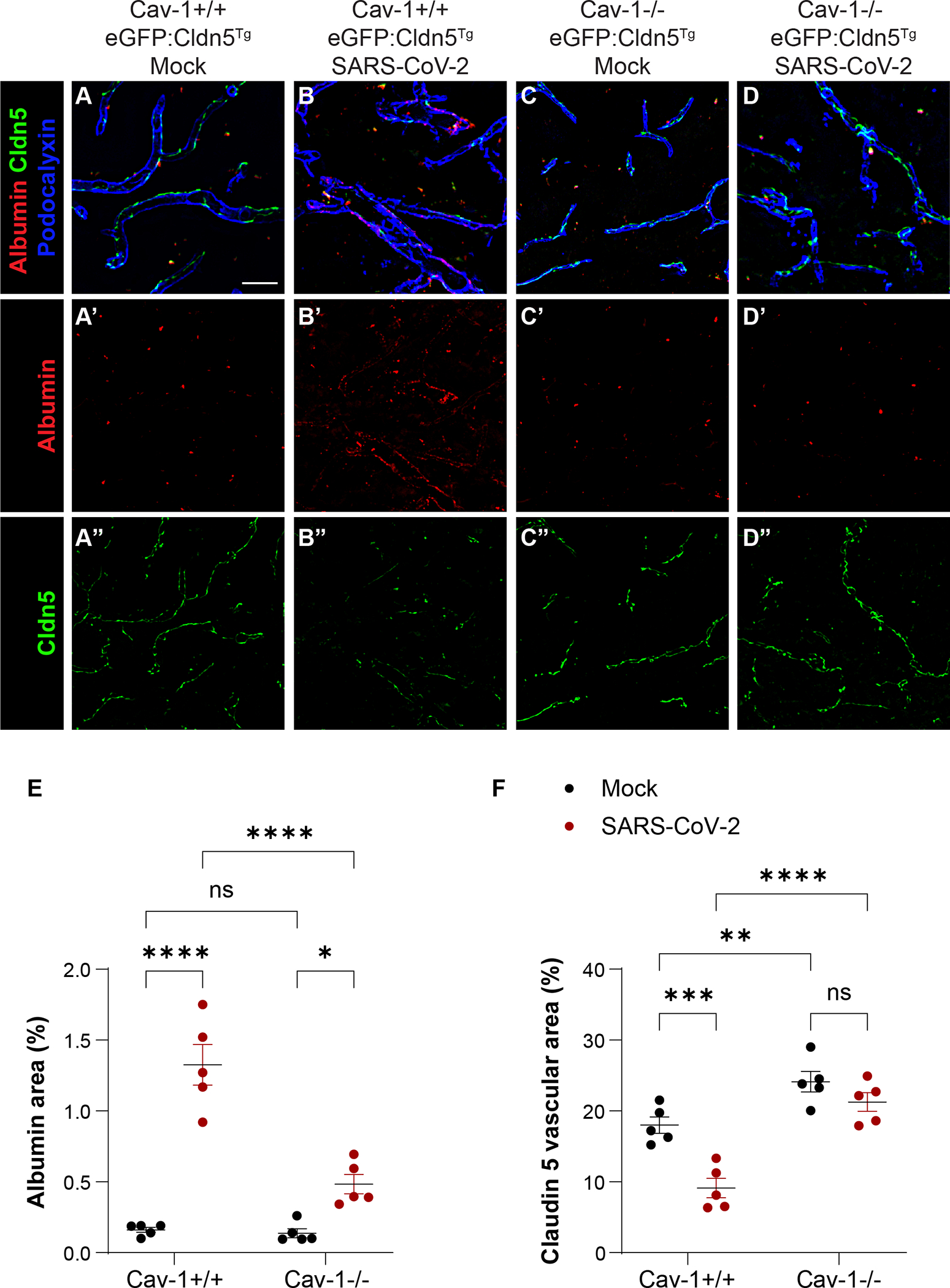
Cav-1 dysregulates features of transcellular and paracellular BBB integrity in SARS-CoV-2 infection. A-D) Micrographs depict brain distribution of intravenously injected Alexa594-Albumin (red), Claudin 5 tight junctions (green), and immunodetection of the endothelial protein podocalyxin (blue) in hippocampus of eGFP-Cldn5^Tg/−^Cav-1^+/+^ and eGFP-Cldn5^Tg/−^Cav-1^−/−^ mice euthanized 5 DPI with mock or with SARS-CoV-2. Brain accumulation of intravenously injected Alexa594-Albumin, a primarily transcellular BBB cargo, in SARS-CoV-2 infected Cav-1^+/+^ mice (B′) but not in SARS-CoV-2 infected Cav-1^−/−^ mice (D′) suggests Cav-1 contributes to disruption of the transcellular BBB barrier in SARS-CoV-2 infection. Decreased cerebrovascular Claudin-5 coverage in SARS-CoV-2 infected Cav-1^+/+^ mice (B″) but not in SARS-CoV-2 infected Cav-1^−/−^ mice (D″) suggests Cav-1 contributes to disruption of the paracellular BBB barrier in SARS-CoV-2 infection. Scale bar, 20 μm. *n* = 5 mice/group, with three sections analyzed per mouse. E) Quantification of Alexa594-Albumin positive area in the hippocampus of eGFP-Cldn5^Tg/−^Cav-1^+/+^ and eGFP-Cldn5^Tg/−^Cav-1^−/−^ mice at 5 DPI with mock or with SARS-CoV-2. Two way ANOVA demonstrated significant effect of genotype [F_(1, 16)_ = 28.30, *p* < 0.0001], infection [F_(1, 16)_ = 86.11, *p* < 0.0001], and genotype*infection interaction [F_(1, 16)_ = 25.32, *p* = 0.0001]. SARS-CoV-2 infection increased albumin extravasation in Cav-1^+/+^ mice (*p* < 0.0001) and in Cav-1^−/−^ mice (*p* < 0.05). Sidak’s multiple comparisons test revealed significantly greater albumin extravasation in Cav-1^+/+^ mice with SARS-CoV-2 as compared to Cav-1^−/−^ mice with SARS-CoV-2 (*p* < 0.0001). F) Quantification of Claudin-5 vascular coverage in the hippocampus of eGFP-Cldn5^Tg/−^Cav-1^+/+^ and eGFP-Cldn5^Tg/−^Cav-1^−/−^ mice at 5 DPI with mock or with SARS-CoV-2. Podocalyxin immunoreactivity was used to define endothelial area. Graph presents fraction of endothelial area positive for Claudin 5. Two way ANOVA demonstrated significant effect of genotype [F_(1, 16)_ = 25.15, *p* < 0.0001], infection [F_(1, 16)_ = 58.58, *p* < 0.0001], and genotype*infection interaction [F_(1, 16)_ = 4.745, *p* = 0.0447]. Post-hoc analysis with Sidak’s multiple comparisons test revealed significant decreases in Claudin 5 coverage in Cav-1^+/+^ mice with SARS-CoV-2 compared to Cav-1^+/+^ mice with mock infection (*p* < 0.0005). Claudin 5 coverage was not decreased by SARS-CoV-2 infection in Cav-1^−/−^ mice (*p* = 0.226). Cav-1^−/−^ mice had significantly greater Claudin 5 coverage than did Cav-1^+/+^ mice in the mock infection (*p* = 0.005) and in the SARS-CoV-2 infection (*p* < 0.0001) groups.

We next analyzed the effect of SARS-CoV-2 induced upregulation of Cav-1 on transcellular BBB permeability. Albumin can cross endothelial cells in a receptor-mediated endocytosis/transcytosis pathway involving Cav-1 (Hu and Minshall, 2009; Knowland et al., 2014; Lutz et al., 2017; Tiruppathi et al., 2004; Zimnicka et al., 2016). We inoculated 12-month-old Cav-1^+/+^ and Cav-1^−/−^ mice with SARS-CoV-2. To functionally test transcellular BBB permeability, we intravenously injected these mice with albumin conjugated to the fluorophore Alexa594 thirty minutes prior to euthanasia by transcardial perfusion at 5 DPI. We used fluorescence microscopy to assess the hippocampal area positive for Alexa594-Albumin (Fig. 2A–D). We found that SARS-CoV-2 increased BBB permeability to albumin in Cav-1^+/+^ mice but not in Cav-1^−/−^ mice (Fig. 2E). These data indicate that in the MA10 model, SARS-CoV-2 increases transcellular BBB macromolecule permeability in a Cav-1 dependent manner.

We also assessed the effect of SARS-CoV-2 induced upregulation of Cav-1 on tight junctions, a correlate of paracellular BBB integrity. Cav-1 promotes the endocytosis and degradation of junctional proteins in response to inflammatory cytokines (Kronstein et al., 2012; Li et al., 2015; Liu et al., 2012; Marchiando et al., 2010; Nag et al., 2007; Song et al., 2007; Stamatovic et al., 2009). We therefore interrogated if Cav-1 contributes to BBB Claudin-5 degradation in SARS-CoV-2 infection. We used eGFP:Claudin-5^Tg/−^ Cav-1^+/+^ and eGFP:Claudin-5^Tg/−^ Cav-1^−/−^ mice with genetically encoded eGFP-Claudin-5 to facilitate tight junction visualization. To assess tight junction coverage, we used immunostaining for the pan-endothelial protein podocalyxin to define the cerebrovascular area, and measured the fraction of this area positive for eGFP-Claudin-5 (Fig. 2A–D). We found that SARS-CoV-2 caused a significant decrease in tight junction coverage in eGFP:Claudin-5^Tg/−^ Cav-1^+/+^ but not in eGFP:Claudin-5^Tg/−^ Cav-1^−/−^ mice (Fig. 2F). We also observed a correlation between Claudin 5 coverage and albumin permeability (Supplementary Fig. 4), suggesting that transcellular and paracellular permeability are both features of infection. Overall, our data indicate that Cav-1 deficiency protects mice from SARS-CoV-2-induced transcellular and paracellular permeability.

In addition, we noted that cerebrovascular Claudin-5 coverage was significantly greater in mock-infected eGFP:Claudin-5^Tg/−^ Cav-1^−/−^ mice as compared with mock-infected eGFP:Claudin-5^Tg/−^ Cav-1^+/+^ mice (Fig. 2A, C, E). This suggests that Cav-1 contributes to homeostatic cerebrovascular tight junction degradation in advanced age in the absence of disease.

### 2.3. Cav-1 promotes neuroinflammation in SARS-CoV-2 infection

**Fig. 3.**
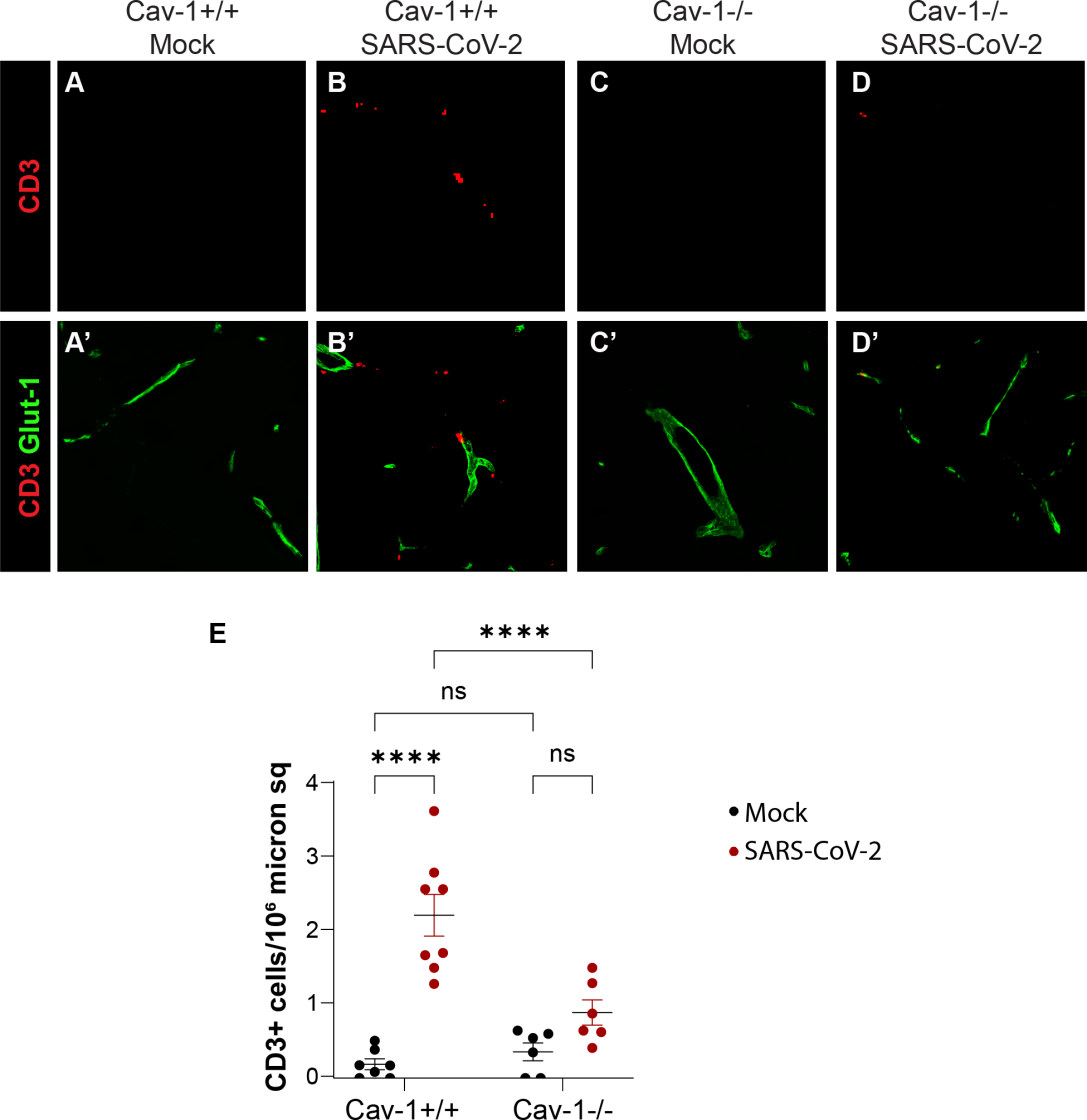
Cav-1 deficiency reduces hippocampal T cell density in SARS-CoV-2 infection. A-D) Immunofluorescence detection of CD3+ T cells (red) and Glut-1+ cerebrovasculature (green) in hippocampal sections of mice euthanized at 5 DPI with SARS-CoV-2. E) Quantification of CD3+ T cells in hippocampal sections. *n* = 6–8 mice/group, with two sections analyzed per mouse. Two-way ANOVA demonstrated significant effect of genotype [F_(1,23)_ = 8.409, *p* = 0.0081], infection [F_(1, 23)_ = 41.25, *p* < 0.0001], and genotype*infection interaction [F_(1, 23)_ = 13.99, *p* = 0.0011]. Tukey’s multiple comparison tests revealed SARS-CoV-2 significantly increased T cells in the hippocampus of Cav-1^+/+^ mice (*p* < 0.001) but not Cav-1^−/−^ mice (*p* = 0.09). There were significantly fewer T cells in hippocampus of Cav-1^−/−^ mice with SARS-CoV-2 as compared to Cav-1^+/+^ mice with SARS-CoV-2 (*p* < 0.01).

Heightened T cell infiltration of the CNS is a consequence of dysregulation of the BBB. We next analyzed T cell infiltration into the hippocampal parenchyma in Cav-1^+/+^ and Cav-1^−/−^ mice infected with MA10 SARS-CoV-2. T cells were increased in SARS-CoV-2 infected Cav-1^+/+^ mice compared to healthy controls (Fig. 3A–B). Importantly, we observed fewer CD3+ T cells in the hippocampus in SARS-CoV-2 infected Cav-1^−/−^ mice compared to infected Cav-1^+/+^ mice (Fig. 3C–E). This suggests that Cav-1 deficiency may offer protection against SARS-CoV-2 neuroinflammation by attenuating T cell migration across the BECs.

**Fig. 4.**
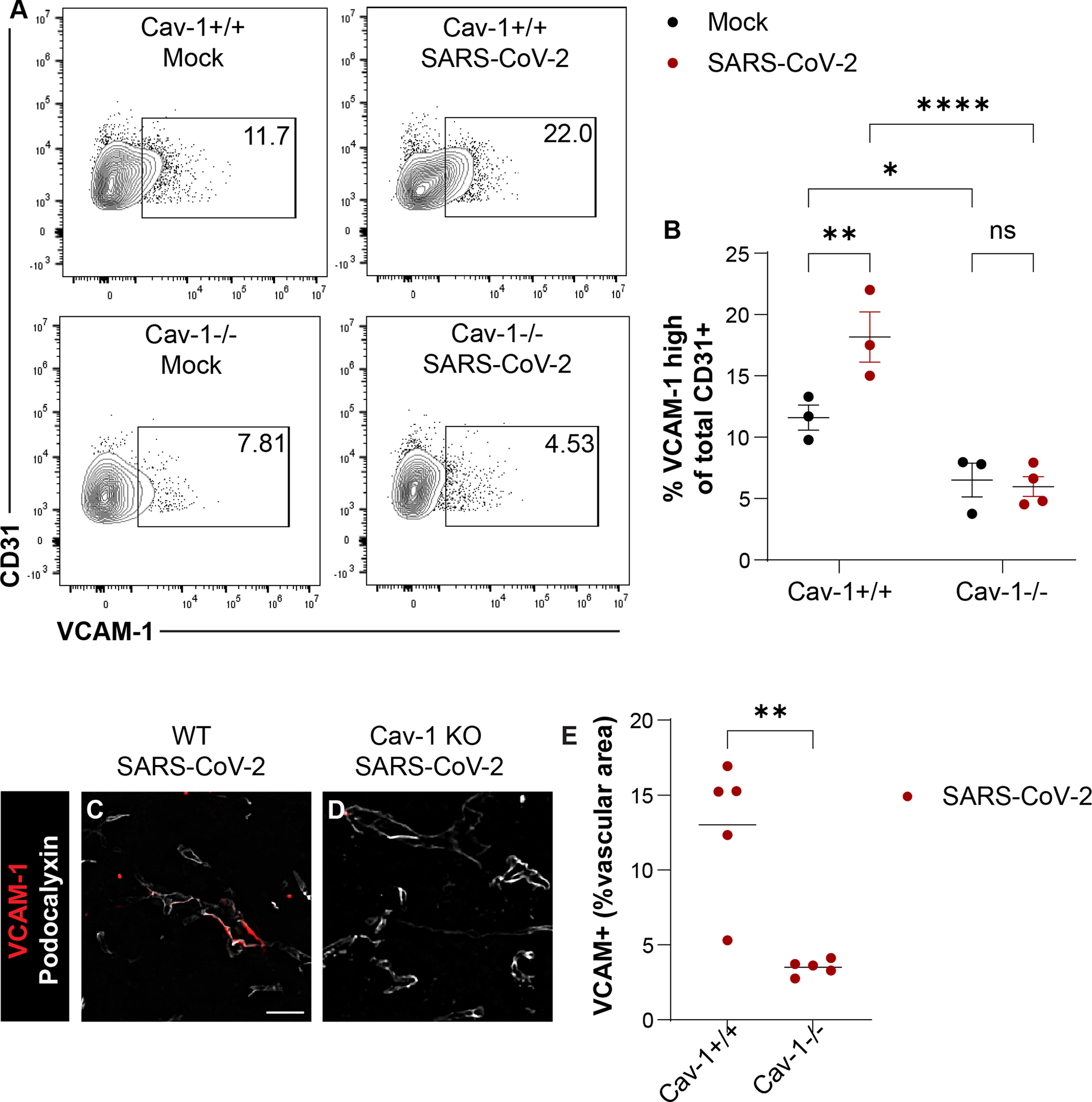
Cav-1 deficiency reduces brain endothelial cell VCAM-1 expression in SARS-CoV-2 infection. A) Flow cytometry of VCAM-1 and CD31 in brain endothelial cells. B) Quantification of VCAM-1^high^ cells as a percent of total CD31+ viable cells. *n* = 3–4 mice/group. Two-way ANOVA demonstrated significant effect of genotype [F_(1, 9)_ = 42.64, *p* = 0.0001], infection [F_(1, 9)_ = 5.188, *p* = 0.0487], and genotype*infection interaction [F_(1, 9)_ = 7.231, *p* = 0.0248]. Tukey’s multiple comparison tests revealed significantly increased BEC VCAM-1 in Cav-1^+/+^ (*p* < 0.01) but not Cav-1^−/−^ mice with SARS-CoV-2. VCAM-1^high^ BEC were significantly fewer in Cav-1^−/−^ mice with SARS-CoV-2 than in Cav-1^+/+^ mice with SARS-CoV-2 (*p* < 0.0001). C-D) Immunofluorescence detection of VCAM-1 (red) and podocalyxin (white) in Cav-1^+/+^ and Cav-1^−/−^ mice at 5DPI SARS-CoV-2. *n* = 5 mice/group, with three sections analyzed per mouse. E) Quantification of VCAM-1+ vascular area. Podocalyxin immunoreactivity was used to define vascular area. There was significantly less VCAM-1+ immunoreactive vascular area in Cav-1^−/−^ mice with SARS-CoV-2 than in Cav-1^+/+^ mice with SARS-CoV-2 (*p* < 0.01, unpaired *t*-test with Welch’s correction for unequal variance).

Cav-1 functions as a scaffolding molecule involved in the membrane expression of leukocyte adhesion molecules (Carman and Springer, 2004; Dragoni et al., 2017; Han et al., 2010; Heemskerk et al., 2016; Hu et al., 2008; Kanters et al., 2008; Lolo et al., 2022; Millan et al., 2006; Wu et al., 2016; Xu et al., 2013). We therefore investigated if Cav-1 might influence brain endothelial cell vascular cell adhesion molecule 1 (VCAM-1) expression in SARS-CoV-2 infection. We analyzed VCAM-1 expression on brain endothelial cells isolated from SARS-CoV-2 infected mice at 5 DPI. The percentage of brain endothelial cells highly expressing VCAM-1 was significantly increased in MA10 SARS-CoV-2 infected Cav-1^+/+^ mice but not Cav-1^−/−^ mice (Fig. 4A–B). Similarly, by immunostaining, Cav-1^−/−^ mice with SARS-CoV-2 infection had less VCAM-1 positive cerebrovascular area than did Cav-1^+/+^ mice with SARS-CoV-2 infection (Fig. 4C–E). These data indicate that Cav-1 contributes to brain endothelial cell immune activation in SARS-CoV-2 infection.

**Fig. 5.**
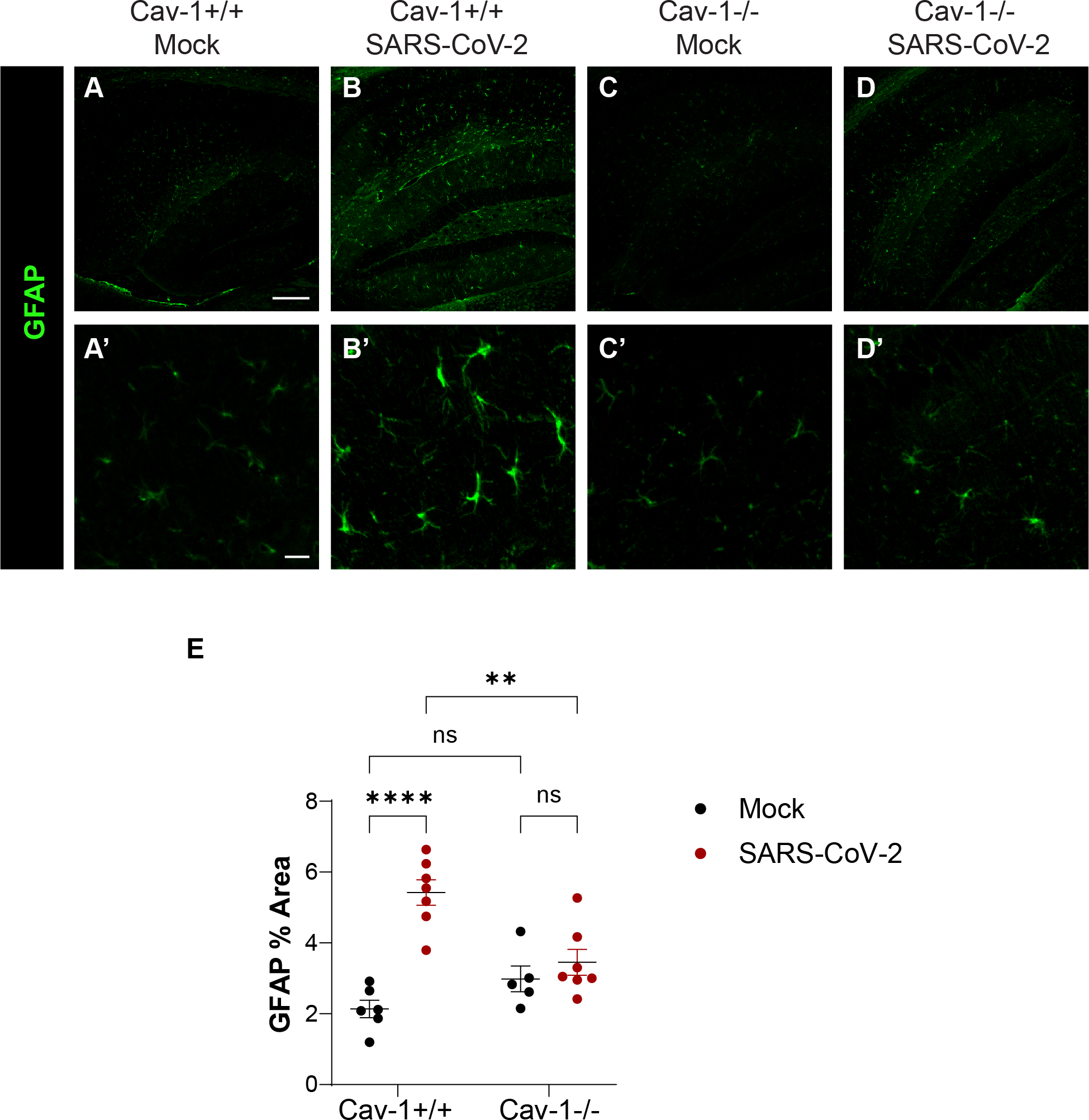
Cav-1 deficiency reduces GFAP expression induced by SARS-CoV-2 infection. A-D) Immunofluorescence detection of GFAP in the hippocampus of Cav-1^+/+^ and Cav-1^−/−^ mice 5 DPI with mock or SARS-CoV-2. Scale bar in A-D is 200 μm. Scale bar in A’-D′ is 20 μm. E) Quantification of area immunoreactive for GFAP in the hippocampus. n = 6–7 mice/group, with two sections analyzed per mouse. Two way ANOVA demonstrated significant effect of infection [F_(1,21)_ = 29.33, *p* < 0.0001] and infection*genotype interaction [F_(1, 21)_ = 16.52, *p* = 0.0006]. Sidak’s multiple comparisons test revealed that SARS-CoV-2 increased GFAP immunoreactivity in Cav-1^+/+^ (*p* < 0.0001) but not Cav-1^−/−^ (*p* = 0.83) mice. GFAP was significantly lower in Cav-1^−/−^ mice with SARS-CoV-2 than in Cav-1^+/+^ mice with SARS-CoV-2 (*p* = 0.0013).

A consequence of BBB permeability to macromolecules and immune cells can include inflammatory changes in glial cells. We next investigated expression of GFAP in SARS-CoV-2 infection. We observed that GFAP immunoreactive area was significantly upregulated in Cav-1^+/+^ mice infected with SARS-CoV-2 but not in Cav-1^−/−^ mice infected with SARS-CoV-2 (Fig. 5A–E). This suggests that in SARS-CoV-2 infection, upregulation of cerebrovascular Cav-1 and resulting BBB permeability contribute to reactive changes in astrocytes.

Previous studies indicate that brain endothelial cells undergo changes in SARS-CoV-2 infection including cell death (Soung et al., 2022). We investigated endothelial cell apoptosis by immunostaining for Caspase 3. We observed that SARS-CoV-2 induced brain endothelial cell apoptosis to a similar extent in Cav-1^+/+^ and in Cav-1^−/−^ mice (Supplementary Fig. 5A–E).

### 2.4. Cav-1 promotes neurological signs of disease in SARS-CoV-2 infection

**Fig. 6.**
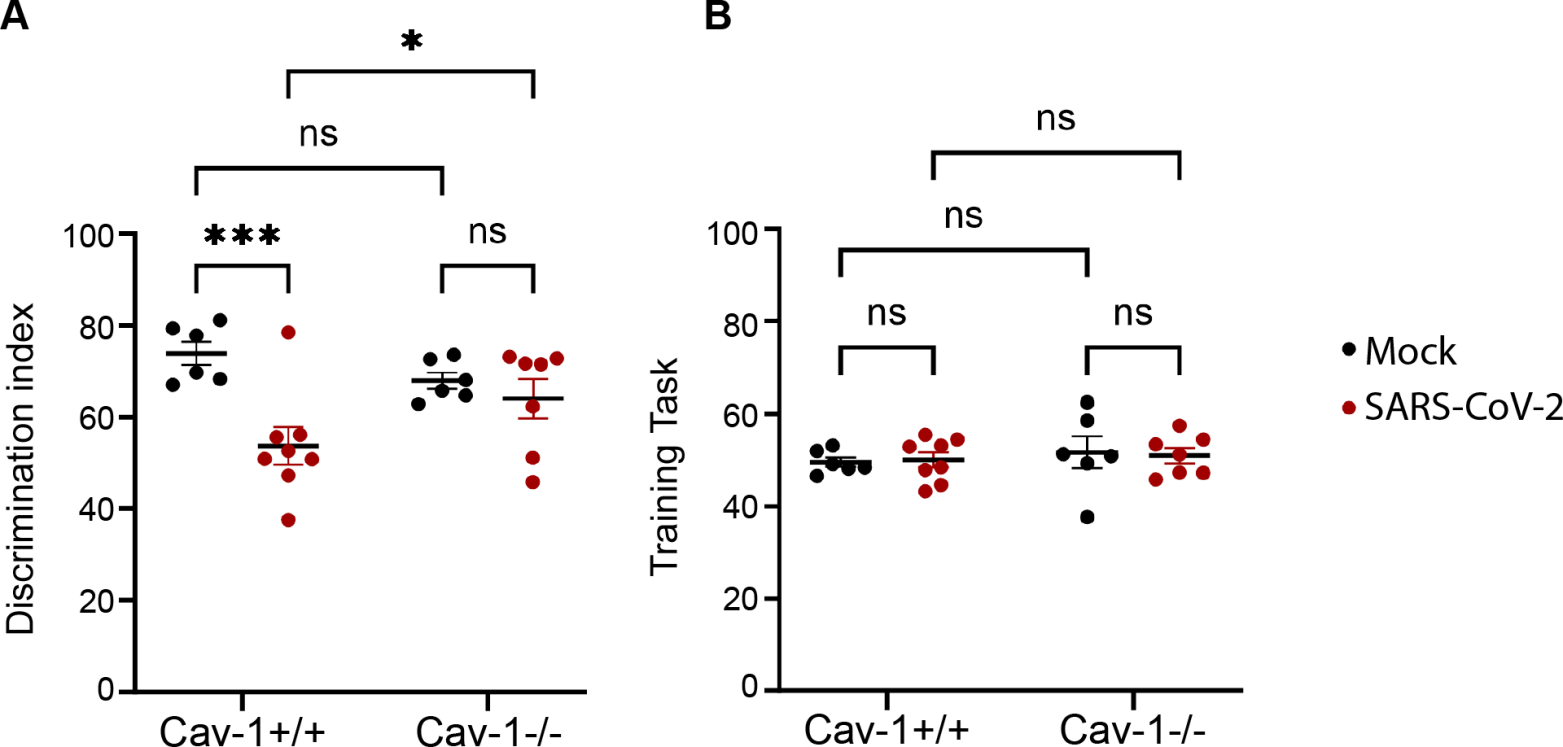
Cav-1 deficiency reduces short term learning and memory impairment in SARS-CoV-2 infection. A) Novel object discrimination index during novel object recognition testing at 5 DPI with SARS-CoV-2. n = 5–8 mice/group. Two-way ANOVA demonstrated significant effect of infection [F_(1, 23)_ = 11.15, *p* = 0.0028], and genotype*infection interaction [F_(1, 23)_ = 5.103, *p* = 0.0337]. Tukey’s multiple comparison test revealed significant decrease in novel object discrimination in Cav-1^+/+^ (*p* < 0.001) but not Cav-1^−/−^ mice with SARS-CoV-2. Cav-1^−/−^ mice with SARS-CoV-2 performed significantly better than did Cav-1^+/+^ mice with SARS-CoV-2. B) Object discrimination index measured during the training task demonstrates unbiased exploration during the training phase in all groups.

Neurological impairment can accompany acute SARS-CoV-2 infection (Spudich and Nath, 2022). The extent to which BBB inflammation influences neurological deficits in COVD-19 is unclear. We therefore tested whether Cav-1 deficiency offers protection from SARS-CoV-2 induced cognitive impairment. We utilized a novel object recognition task to measure learning and memory related to hippocampal function. Cav-1^−/−^ mice have some age-dependent neurobehavioral abnormalities, including spatial learning deficit and center avoidance (Gioiosa et al., 2008; Trushina et al., 2006). Nonetheless, in our study, mock infected Cav-1^−/−^ mice had similar novel object recognition as did mock infected Cav-1^+/+^ mice (Fig. 6A). In Cav-1^+/+^ mice, SARS-CoV-2 respiratory infection significantly impaired the ability to discriminate between known and unknown objects (Fig. 6A). Importantly, Cav-1 deficiency mitigated this effect of SARS-CoV-2 on learning and memory (Fig. 6A). As expected, no object preferences were observed during the training task in either genotype (Fig. 6B). Overall, our data suggest that Cav-1 upregulation on BECs promotes neuroinflammation and neurological deficits in COVID-19 by modifying BBB function.

## 3. Discussion

COVID-19-associated neurological impairment, cerebrovascular damage, and neuroinflammation are well-documented (Fernández-Castañeda et al., 2022; Hernández-Fernández et al., 2020; Hosp et al., 2021; Lee et al., 2021; Lee et al., 2022; Schwabenland et al., 2021; Soung et al., 2022; Spudich and Nath, 2022; Thakur et al., 2021). In this work, we sought to identify the role of BBB dysfunction in SARS-CoV-2 induced neuroinflammation and cognitive impairment, and sought to determine whether deficiency in Cav-1 can offer protection.

We observed that brain endothelial cell Cav-1 is upregulated by SARS-CoV-2 respiratory infection. Our observation is consistent with recent reports of increased Cav-1 in the forebrain of COVID-19 decedents (Green et al., 2022; Premkumar and Sajitha, 2023). In our data, the upregulation of Cav-1 in the cerebrovasculature induced by SARS-CoV-2 was linked to worse BBB permeability, T-lymphocyte infiltration, gliosis, and cognitive impairment, whereas these signs of disease were attenuated in SARS-CoV-2 infected mice genetically deficient in Cav-1. These data suggest a potential role for Cav-1 in BBB permeability in COVID-19 neuroinflammation.

Cav-1 contributes to transcellular and paracellular BBB permeability. Cav-1 functions in endocytosis and transcytosis of proteins and cells from the luminal to the abluminal endothelial cell surface (Jones and Minshall, 2020; Ohi and Kenworthy, 2022; Parton, 2018; Pol et al., 2020; Zimnicka et al., 2016). In development, transcellular BBB permeability is suppressed by pericyte and astrocyte regulation of Cav-1 (Andreone et al., 2017; Armulik et al., 2010; Ayloo et al., 2022; Chow and Gu, 2017; Daneman et al., 2010; Guérit et al., 2020), whereas caveolar transcytosis can also be induced in the mature BBB by endothelial autonomous mechanisms (Chang et al., 2022; Liebner et al., 2018; Pandit et al., 2020; Villaseñor et al., 2017). This transcellular activity is heightened by inflammation and advanced age (Carman and Springer, 2004; Hu et al., 2008; Marchiando et al., 2010; Millan et al., 2006; Yang et al., 2020). In our data, SARS-CoV-2 infection induced transcellular BBB permeability to albumin by upregulated brain endothelial Cav-1. Furthermore, Cav-1 contributes to the membrane removal, recycling, and degradation of tight junction molecules such as Claudin-5, especially in the context of inflammatory cytokines (Li et al., 2015; Liu et al., 2012; Marchiando et al., 2010; Stamatovic et al., 2009). Because tight junctions are a critical component of paracellular BBB function, heightened Cav-1 activity indirectly promotes paracellular BBB permeability. In our data, SARS-CoV-2 infection decreased cerebrovascular tight junction coverage in a Cav-1 dependent way, which we posit is due to caveolar endocytosis and degradation of BBB tight junctions. Future studies could intravitally probe rates of caveolar endocytosis of BBB junctional proteins and functionally probe paracellular BBB permeability using small and large molecular weight fluorescent tracers in infected mice and further define the downstream mechanisms by which Cav-1 contributes to loss of tight junctions in this model. Our findings implicate Cav-1 as a regulator of multiple aspects of BBB leakage in SARS-CoV-2 infection.

Caveolin-1 can promote adhesion and migration of leukocytes across the BBB in part by influencing endothelial expression and distribution of leukocyte adhesion molecules (Wu et al., 2016). Here, we found that Cav-1 contributes to cerebrovascular expression of VCAM-1 and T lymphocyte infiltration of the hippocampus in SARS-CoV-2. Our findings fit within a larger context of brain endothelial hyperinflammatory responses and systemic inflammation in COVID-19 (Bonetto et al., 2022; Frere et al., 2022; Pilotto et al., 2021; Rutkai et al., 2022; Soung et al., 2022).

Consequentially, we observed that SARS-CoV-2-induced hippocampal neuroinflammation and cognitive impairment were attenuated in Cav-1 deficient mice. Perivascular leukocyte cuffing, gliosis, and neuronophagia are observed in COVID-19 and its animal models (Amruta et al., 2022; Lee et al., 2021; Lee et al., 2022; Matschke et al., 2020; Schwabenland et al., 2021; Soung et al., 2022; Spudich and Nath, 2022; Thakur et al., 2021). Interestingly, extensive perivascular leukocyte cuffing reportedly only occurs in a subset of COVID-19 decedents, suggesting that individual risk factors modify CNS consequences of infection (Agrawal et al., 2022; Thakur et al., 2021). Indeed, advanced age, preexisting vascular disease, and other conditions causing heightened cerebrovascular activation, which might include Cav-1 or other regulators of BBB permeability, increase risk of severe neurologic outcome of SARS-CoV-2 infection (Adesse et al., 2022; Monje and Iwasaki, 2022; Sullivan and Fischer, 2021; Vanderheiden and Klein, 2022; Vavougios et al., 2022). Overall, these data suggest a potential therapeutic value of targeting BBB permeability to improve disease outcomes in SARS-CoV-2 infection.

No animal model can fully recapitulate complex human diseases. Advantages of the SARS-CoV-2 MA10 infectious model include that because the virus binds to mouse ACE2, it results in infection of those cells that have endogenous ACE2 expression. In mice, like in humans, expression of ACE2 is robust in the alveolar epithelium and alveolar type II (AT2) cells of the respiratory tract. Consequently, AT2 cells are the predominant population of infected cells in the respiratory tract in mice infected with MA10 and in humans infected with the SARS-CoV-2 ancestral (WA-1) strain. However, the cellular patterns of ACE2 expression and MA10 infection in mice may not completely match infection by SARS-CoV-2 in humans; indeed the cellular targets of infection by SARS-CoV-2 in humans may differ based upon viral strain and variant. As such, caution is warranted in generalizing our findings to the human condition.

Because our goal in this study was to define the contribution of the BBB protein Cav-1 to neurobehavior, we focused on the hippocampus as a relevant neuroanatomic structure with readily measurable behavioral outcomes. Nonetheless, we also observed SARS-CoV-2 induced upregulation of vascular Cav-1 in other neuroanatomic regions notable for inflammation in COVID-19, including the olfactory bulb, cortex, and brainstem, and corroborated this finding in endothelial cells isolated from whole brain. Thus, SARS-CoV-2 induced upregulation of Cav-1 in multiple brain regions. Our data raise the key question of whether Cav-1 mediated changes to the BBB in other brain regions impacted by SARS-CoV-2 infection also influence neurobehavioral outcomes of disease. Larger animal models may be better suited for testing the neuroanatomic and mechanistic basis of complex neurobehavioral deficits reflecting neural processes superseding the hippocampus and difficult to recapitulate in mice, such as brain fog, attention deficit, and affective change.

Indeed, although our data supports a central role for Cav-1 in neuroinflammation and cognitive impairment, it is important to note caveats. Importantly, no differences were noted in viral RNA in the lung, systemic hypoxia, or weight loss between Cav-1^+/+^ and Cav-1^−/−^ mice with SARS-CoV-2 infection. Nonetheless, variability was observed within groups in the amount of viral RNA in the lung; variability in the severity of the pulmonary infection, as well as natural variability found in aged mice, may have contributed variability to the experimental results.

In this study, we did not identify the proximal cause of cerebrovascular Cav-1 upregulation in SARS-CoV-2 infection. There are a number potential mechanisms. Brain endothelial cells are not the primary cellular targets of SARS-CoV-2 infection in the respiratory inoculation model. Nonetheless, direct infection of brain EC could have led to dysregulation of Cav-1. However, data are limited to support that idea, especially as Cav-1 is not reported to be upregulated in brain endothelial cells infected with SARS-CoV-2 *in vitro* (Motta et al., 2023; Yang et al., 2022). Nonetheless, SARS-CoV-2 infection is closely associated with production of numerous circulating proinflammatory mediators including viral proteins, cytokines, and activated leukocytes, all of which are anticipated to directly and indirectly impinge upon brain EC homeostasis (Vanderheiden and Klein, 2022). Additional studies will be required to elucidate the molecular mediators between respiratory infection and brain EC dysfunction.

Likewise, we have not yet fully mapped the mediators between elevated cerebrovascular Cav-1 and impaired neurologic function. For example, we have not defined the contribution of Cav-1 to neuronophagia, synaptophagia, or impaired neurogenesis in SARS-CoV-2 infection. Nonetheless, correlations between features of BBB permeability, neuroinflammation, and cognitive impairment are consistent with a model in which Cav-1 mediated BBB permeability leads to neuronal dysfunction.

Because SARS-CoV-2 is a BSL3 pathogen, we were limited in the kinds of neurobehavioral tests we could conduct to assess neuronal function, because of the practical limitations to conducting behavior tests within the confines of the biosafety cabinet in the BSL3 suite.

This study exclusively examined acute infection, so we cannot draw conclusions regarding neurological post-acute sequelae of COVID-19 (neuroPASC). Interestingly, however, neuroPASC correlates to serum markers of BBB leakage (Bonetto et al., 2022; Hanson et al., 2022). These observations suggest the value of future mechanistic studies into BBB Cav-1 dysregulation in neuroPASC.

## 4. Methods

### 4.1. Mice

The study design is depicted in Supplementary Fig. 1. All animal studies were approved by the UIC Animal Care and Use Committee (20–160, 21–051). Wild-type C57Bl/6 and Cav-1 KO (Jackson Laboratory 000664, 004585, respectively) mice were purchased from the Jackson Laboratory and backcrossed 9 generations. eGFP:Cldn5 transgenic mice express Claudin-5 (Cldn5) labeled with enhanced green fluorescent protein to facilitate visualization of BBB tight junctions (Knowland et al., 2014; Lutz et al., 2017). eGFP:Cldn5^Tg/−^Cav1^−/−^ mice were generated by breeding these lines. All the mice used in this study were male, born in house, and maintained in a specific pathogen free vivarium suite. Mice were transferred to Animal Biosafety Level 3 facilities at least 2 days prior to inoculation. Mice were maintained on standard light-dark cycles with *ad libitum* food and water in micro-isolation cages. Mice were assigned numeric codes which were used to track the samples in a blinded fashion during *in vivo* and post-mortem processing, imaging, and analysis.

### 4.2. SARS-CoV-2 inoculation

Mouse adapted SARS-CoV-2 MA10 (Leist et al., 2020) was propagated and titered on Vero-E6 cells expressing ACE2 and TMPRSS2 (ATCC, CRL1586). MA10 was generated by engineering the spike protein to bind to the murine homolog of the viral entry receptor, ACE2, and 10 sequential passages through mice (Leist et al., 2020). Mouse adapted SARS-CoV-2 (MA10) has been previously characterized (Amruta et al., 2022; Leist et al., 2020). Intranasal inoculation with SARS-CoV-2 MA10 infects ACE2-expressing alveolar epithelium and alveolar type II (AT2) cells within the lung, yielding acute respiratory infection (Leist et al., 2020). SARS-CoV-2 MA10 was delivered by intranasal inoculation with 1 × 10^4^ foci-forming units (FFU) MA10 or vehicle (saline) in a volume of 25 μl in animal Biosafety Level 3 facilities. Characteristics of infection are described in Supplementary Fig. 2. Body weight was assessed with a standard postal scale. Arterial oxygenation was assessed with a pulse oximeter fitted with a sensor adapted for the mouse paw (MouseSTAT Jr. Pulse Oximeter & Heart Rate Monitor). Pulse oximetry was conducted on the hind paws. We present oximetry % change (Supplementary Fig. 1D) and absolute values (Supplementary Fig. 1E), because we noted that the baseline oximetry readings were lower than expected. Previous reports indicate that dark skin pigmentation decreases the accuracy of pulse oximetry readings, especially at lower oxygen saturation (Feiner et al., 2007), which might have influenced the oximetry readings in our black-pigmented mice (Supplementary Fig. 2D–E).

### 4.3. Behavioral assays

Behavior tasks were conducted in a dim biosafety cabinet laminar flow hood in the BSL3 facility. Testing arenas were white plastic bins 13 in. × 19 in. (Ikea) with pebbled floor. For novel object recognition (NOR), we first tested a catalog of 10 objects for intrinsic preference. Objects were similar in size (1–2 in. wide, 3–4 in. tall), visually interesting, without smell, and made of easily cleaned non-porous materials, *e.g.* 25 ml suspension flasks filled with pebbles, 50 ml conical tubes filled with corncob bedding, or 3-D printed flagpoles. Neodynium magnets affixed to each object were used to ensure consistent object placement relative to magnets permanently affixed to the underside of the arena. For familiarization, we placed individual mice in an arena containing two suspension flasks and allowed 10 min exploration. Behavior was filmed with an overhead mounted wide-angle webcam (Logitech C920S HD Webcam). Intersession interval was 14 h. For the testing session, mice were reintroduced into the arena containing one suspension flask and one novel object and filmed for 10 min. Objects and field were cleaned with ethanol and dried in between mice. Testing was conducted between 7:00–10:00 AM. Videos were coded and independently scored by two blinded scientists for duration of exploration of each object. Preference was calculated as (*sec* investigating novel object)/(sec investigating any object)*100. 50% indicates no preference.

### 4.4. Histology and blood-brain barrier

Mice were euthanized 5 days after infection with SARS-CoV-2. For Cohorts A and B, 12-month-old Cav-1^+/+^ and Cav-1^−/−^ mice were perfused with PBS with a peristaltic pump. Brains were fixed in 4% paraformaldehyde for 72 h at room temperature and paraffin embedded. Heat induced antigen retrieval (10 mM Tri‑sodium citrate [dihydrate], 0.05% Tween-20, pH 6.0) was *>*40 min at 98 °C. Slides were blocked with 5% normal serum, 0.1% Triton-X 100. Primary antibodies incubated overnight at 4 °C at 1:100 included Caveolin-1 (Invitrogen PA5–17447), CD3 (Cell Signaling 78588), GFAP (Sigma HPA056030), Caspase 3 (Abcam ab32499), Glut1 (Abcam ab40084), and Podocalyxin (R&D Systems AF1556, at 1:1000). Alexa-fluorophore conjugated secondary antibodies (Invitrogen) were incubated 2 h at 22 °C. Mounting medium contained DAPI (Vectashield Vibrance). Microscopy was conducted using Zeiss LSM880 or Leica DMI8 microscopes. At least two brain sections per mouse were imaged. Quantification was performed using FIJI (NIH).

For Cohort C, 12-month-old eGFP:Claudin-5^Tg/−^ Cav-1^+/+^ and eGFP: Claudin-5^Tg/−^ Cav-1^−/−^ mice received intravenous injection of 100 μl of 1% *w*/*v* Alexa594:Albumin (Invitrogen A13101) reconstituted in sterile PBS. Thirty minutes was allowed for albumin extravasation. Mice were then subjected to transcardial perfusion with PBS and 4% paraformaldehyde. Brains were post-fixed for 3 h at 4 °C, cryoprotected in 30% sucrose, and embedded in OCT. Cryosections were prepared at 20 μm. Antigen retrieval was not conducted. Slides were blocked with 5% normal serum, 0.1% Triton-X 100, and incubated with antibodies to VCAM-1 (Invitrogen 14–1061–85 at 1:100) and podocalyxin (R&D Systems AF1556 at 1:1000) prior to Alexa-conjugated secondary antibodies, mounting, and microscopy as above.

### 4.5. Endothelial cell isolation

Mice were transcardially perfused with PBS 5 days after infection. Brain microvascular endothelial cells were isolated using gradient centrifugation in 25% BSA (Marottoli et al., 2021). Microvessels were dissociated with collagenase/dispase (Millipore Sigma 10269638001) and DNase (Worthington LK003172) for 1 h in 37 °C water bath and passed through 100 μm cell strainer (PluriSelect USA 43–10100–60). Dissociated microvascular cells were stained for flow cytometric analysis.

### 4.6. Flow cytometry

Endothelial cells isolated from brain from SARS-CoV-2 infected mice were stained with fixable viability dye (Zombie Green, Biolegend 423111) and blocked with Fc receptor blockade (anti-mouse CD16/32, Biolegend 101319) followed by incubation with anti-CD31 antibody (BD Biosciences 561073) and anti-VCAM-1 antibody (Biolegend 105719). Fixation and permeabilization (Biolegend 421403) was followed with an antibody against Cav-1 (Cell Signaling 31411S). Stained cells were analyzed with a Beckman CytoFLEX S flow cytometer.

### 4.7. Statistics

Statistics were conducted using GraphPad Prism 10. Pairwise comparisons used Student’s *t*-test, with Welch’s correction when variances were unequal. Grouped data were tested with two-way ANOVA; significant interactions were compared by Sidak’s multiple comparisons test.

## Supplementary Material

Supplementary Figures and Text

Supplementary Figure 1. Study design.

Supplementary Figure 2. Features of SARS-CoV-2 MA10 infection.

Supplementary Figure 3. Representative micrographs of immunostaining for Cav-1.

Supplementary Figure 4. Correlations exist between features of SARS-CoV-2 infection.

Supplementary Figure 5. Endothelial cell Caspase 3 immunoreactivity is increased in SARS-CoV-2 infection.

## Data Availability

Data will be made available on request.
