# Supplementary material for: Caveolin-1 mediates blood-brain barrier permeability, neuroinflammation, and cognitive impairment in SARS-CoV-2 infection
Source: J Neuroimmunol. Author manuscript; Available in PMC 2024 Jun 28. (PMC11212674; doi:10.1016/j.jneuroim.2024.578309)
Supplement: Supplementary Figures and Text [file NIHMS1998421-supplement-Supplementary_Figures_and_Text.pdf]

Manuscript Number

JNIMM-D-23-00520

Title

Loss of Caveolin-1 protects against SARS-CoV-2-induced blood-brain barrier permeability, neuroinflammation, and cognitive impairment

Authors

Troy N. Trevino[1], Ali A. Almousawi[1], KaReisha F. Robinson[1], Avital B. Fogel[1], Jake Class[2], Richard D. Minshall[3], Leon M. Tai[1], Justin M. Richner[2], Sarah E. Lutz[1]

Departments of Anatomy and Cell Biology[1], Microbiology and Immunology[2], and Anesthesiology[3], University of Illinois at Chicago College of Medicine

Corresponding author:

Sarah E. Lutz

Email: selutz@uic.edu

Phone: 312-355-2499

Address: 909 South Wolcott Avenue, College of Medicine Research Building, Room 7093, MC512, University of Illinois at Chicago College of Medicine, Chicago IL 60612, USA.

Supplementary Figures and Legends

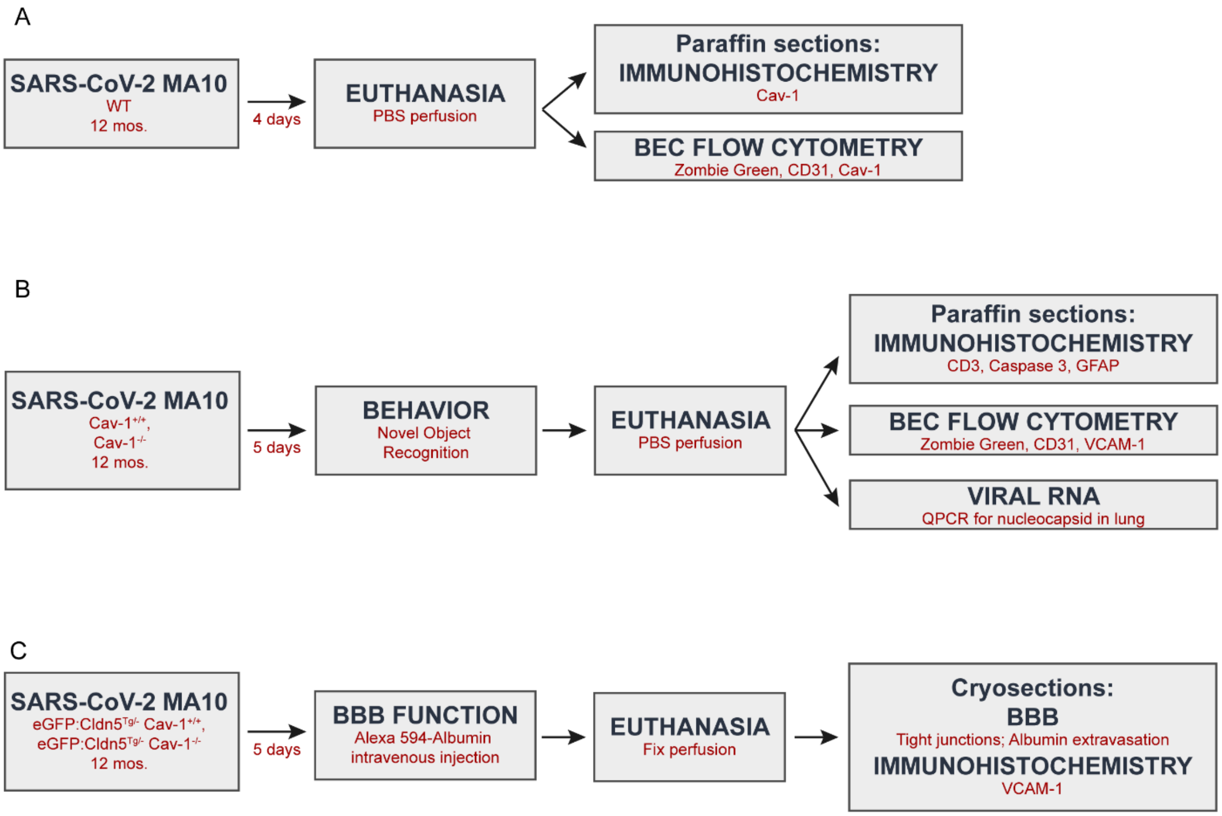

Supplementary Figure 1. Study design. A) Cohort A was an initial study of SARS-CoV-2 MA10 infection in 12-month-old wild type (WT) mice. Mice were euthanized and transcardially perfused with PBS at 4 days post inoculation (DPI). Half brain was immersion fixed in paraformaldehyde, processed, embedded in paraffin, and sectioned for Cav-1 IHC. Half brain was used for brain endothelial cell isolation by gradient centrifugation through 25% BSA (see methods) for flow cytometry. B) Cohort B was designed to assess inflammation. Mice were WT (Cav-1$^{+/+}$) and Cav-1 KO (Cav-1$^{-/-}$) mice, aged 12 months. Mice were used for novel object recognition testing before euthanasia by transcardial perfusion with PBS at 5 DPI. Half brain was prepared for paraffin sections for IHC for CD3, caspase 3, and GFAP. Half brain was used for brain endothelial cell isolation and flow cytometry. Lung was used for Q-PCR to quantify SARS-CoV-2 nucleocapsid RNA. C) Cohort C was designed to address the BBB. Mice had genetically encoded fluorescent tight junction protein, eGFP:Claudin5 (eGFP:Cldn5$^{Tg/-}$) and were either Cav-1$^{+/+}$ or Cav-1$^{-/-}$. Mice were 12 months old and euthanized at 5 DPI. Mice received intravenous injection of fluorescent albumin (Alexa594-Albumin) thirty minutes before euthanasia. Mice were then transcardially perfused with PBS and then with 4%

paraformaldehyde. Cryosections were prepared for quantification of tight junctions, albumin extravasation, and immunofluorescent detection of VCAM-1.

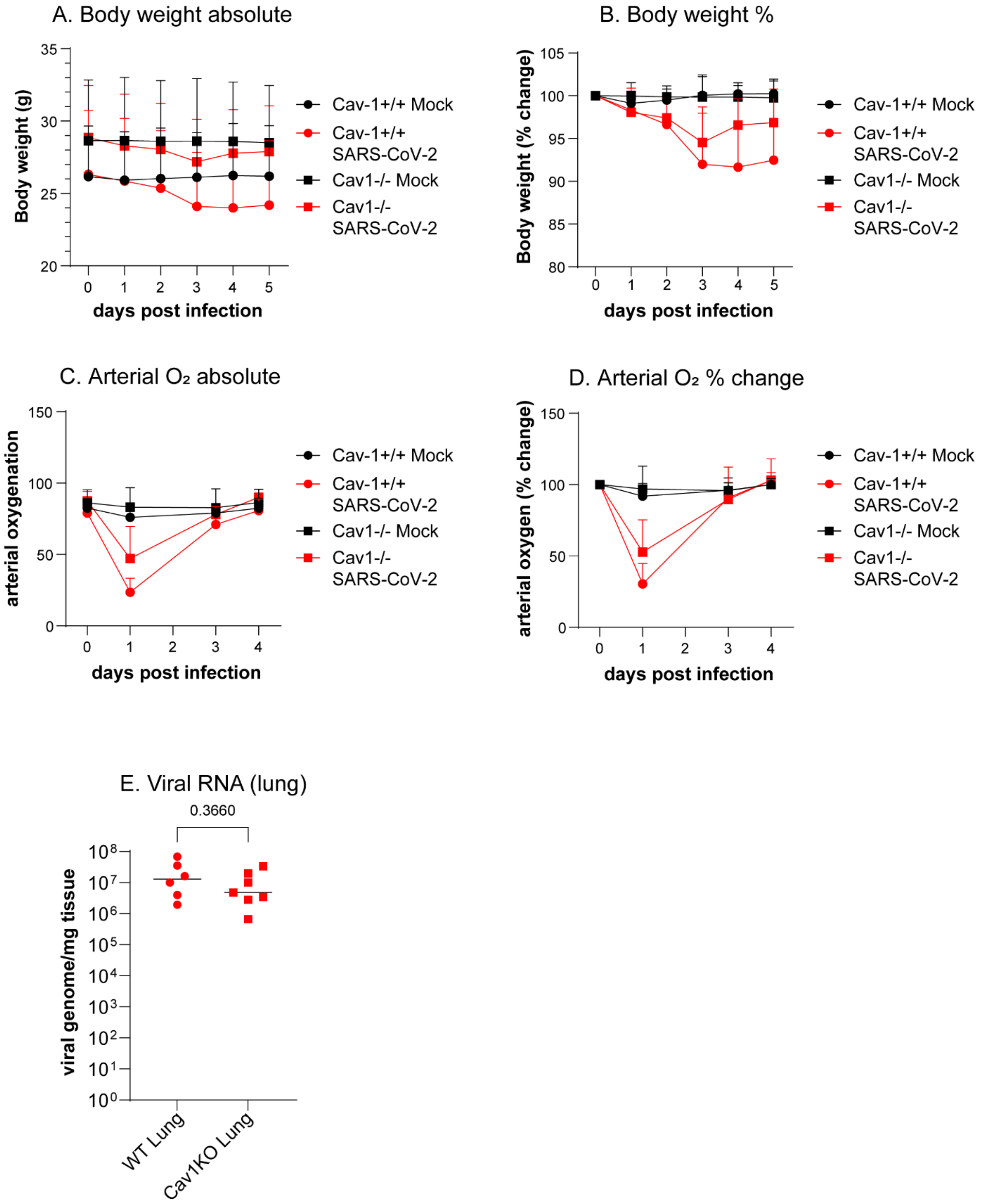

Supplementary Figure 2. Features of SARS-CoV-2 MA10 infection. A) Body weight of mice after intranasal inoculation with vehicle (mock infection) or with SARS-CoV-2

MA10. Two-way RM ANOVA with Geisser-Greenhouse's correction for unequal variance of differences demonstrated significant effect of time [$F_{(1.803, 68.52)}$ = 13.80, $p < 0.0001$] and for time*genotype interaction [$F_{(15, 190)}$ = 6.125, $p < 0.0001$]. No significant differences between groups were noted in Tukey's multiple comparisons test. n = 10-12 mice/group. B) Weight loss expressed as percent change from initial weight for each mouse. n=10-12 mice/group. Two-way RM ANOVA with Geisser-Greenhouse's correction for unequal variance of differences demonstrated significant effect of time [$F_{(1.869, 71.03)}$ = 13.23, $p <$ 0.0001], group [$F_{(3, 38)}$ = 7.439, $p = 0.0005$], and time*group interaction [$F_{(15, 190)}$ = 6.571, $p < 0.0001$]. Tukey's multiple comparisons test revealed significant % weight loss for Cav-$1^{+/+}$ SARS-CoV-2 versus Cav-$1^{+/+}$ Mock at 3DPI ($p < 0.01$) and 4DPI ($p < 0.05$), and for Cav-$1^{-/-}$ SARS-CoV-2 versus Cav-$1^{-/-}$ mock at 3DPI ($p < 0.01$). No significant differences in % body weight loss were observed between Cav-$1^{+/+}$ SARS-CoV-2 and Cav-$1^{-/-}$ SARS-CoV-2 at any day. C) Arterial oxygenation values recorded with a handheld pulse oximeter (see Methods). N=4-5 mice per group. Two-way RM ANOVA with Geisser-Greenhouse's correction for unequal variance of differences demonstrated significant effect of time [$F_{(1.769, 24.76)}$ = 39.16, $p < 0.0001$], group [$F_{(3, 14)}$ = 7.557, $p = 0.0030$], and time*group interaction [$F_{(9, 42)}$ = 9.575, $p < 0.0001$]. Tukey's multiple comparisons test revealed significant differences between Cav-$1^{+/+}$ SARS-CoV-2 versus Cav-$1^{+/+}$ mock at 1DPI ($p < 0.01$). No significant differences were observed between Cav-$1^{+/+}$ SARS-CoV-2 and Cav-$1^{-/-}$ SARS-CoV-2 ($p = 0.25$ at 1DPI). D) Arterial oxygenation percent change from baseline. Two-way RM ANOVA with Geisser-Greenhouse's correction for unequal variance of differences demonstrated significant effect of time [$F_{(1.689, 23.65)}$ = 39.13, $p <$ 0.0001], group [$F_{(3, 14)}$ = 6.657, $p = 0.0051$], and time*group interaction [$F_{(9, 42)}$ = 9.965, p < 0.0001]. Tukey's multiple comparisons test revealed significant differences between Cav-$1^{+/+}$ SARS-CoV-2 versus Cav-$1^{+/+}$ mock at 1DPI ($p < 0.01$) and between Cav-$1^{-/-}$ SARS-CoV-2 versus Cav-$1^{-/-}$ mock at 1DPI ($p < 0.05$). No significant differences were observed between Cav-$1^{+/+}$ SARS-CoV-2 and Cav-$1^{-/-}$ SARS-CoV-2 ($p = 0.34$ at 1DPI). E) Quantification of SARS-CoV-2 nucleocapsid RNA by QPCR in the lower left lobe of the lung at 5DPI. No significant difference was noted between Cav-$1^{+/+}$ and Cav-$1^{-/-}$ (p = 0.37, two tailed Mann Whitney U test).

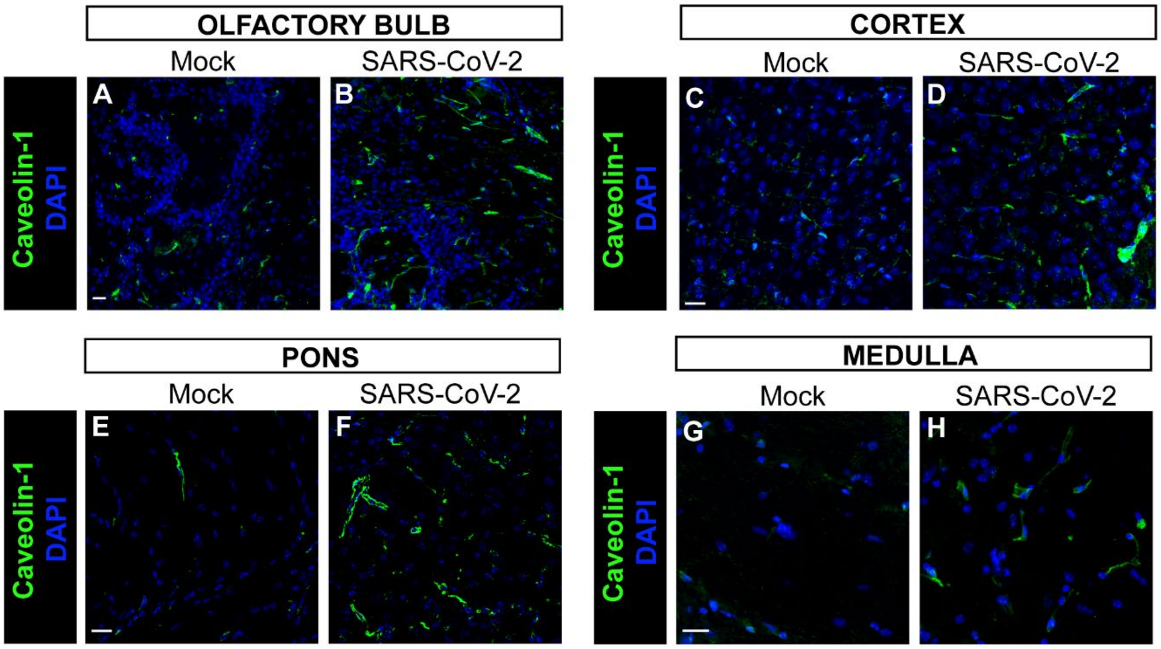

Supplementary Figure 3. Representative micrographs of immunostaining for Cav-1. A-H) Immunostaining for Cav-1 (green) and nuclear counterstain DAPI (blue) in brains of wild-type mice infected with mock or with SARS-CoV-2, at 4DPI. Depicted are A-B) olfactory bulb glomerular layer; C-D) premotor cortex; E-F) pons; G-H) medulla. Scale bars are 20 μm.

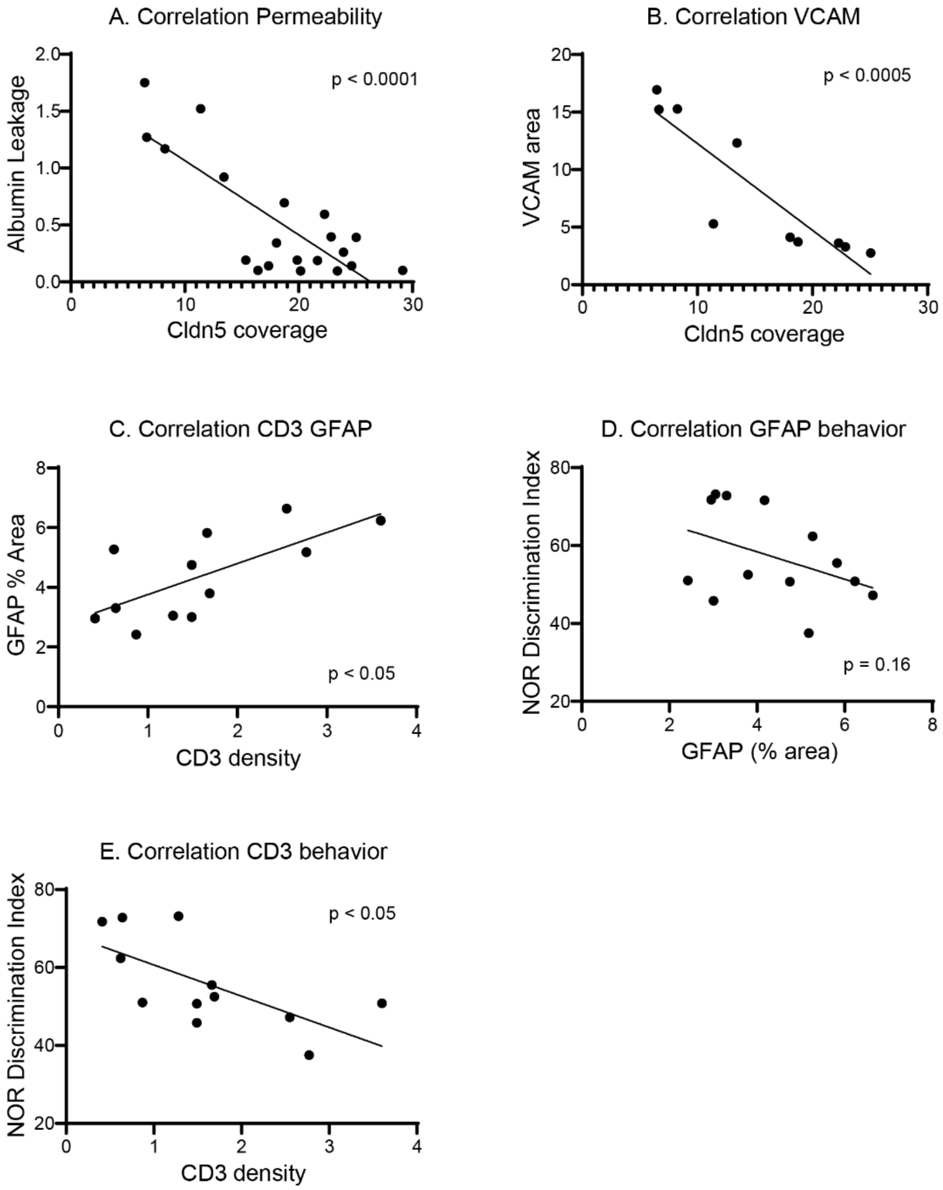

Supplementary Figure 4. Correlations exist between features of SARS-CoV-2 infection. Pearson's correlation analysis was conducted for outcome measurements in Cohort C (A-B) and for outcome measurements in Cohort B (C-E). A) An inverse correlation between Cldn5 vascular coverage and Alexa594-Albumin leakage into the hippocampal parenchyma ($p < 0.0001$; r = -0.8, R squared = 0.64) suggests that features of transcellular and paracellular BBB are both impacted by infection. B) An inverse correlation between Cldn5 vascular coverage and VCAM-1 vascular coverage (*p* <

0.0005; r = -0.896; R squared = 0.80) suggests that changes to the structure of the BBB occur in correlation with changes in endothelial immune activation. C) There was a correlation between CD3+ T cell density in the hippocampus and GFAP immunoreactivity in the hippocampus ($p < 0.05$; r = 0.690; R squared = 0.477). D) No significant correlation between GFAP in the hippocampus and performance on the novel object recognition task ($p = 0.16$; r = -0.410; R squared = 0.168). E) There was an inverse correlation between CD3+ T cell density in the hippocampus and performance on the novel object recognition task ($p < 0.05$; r = -0.666; R squared = 0.4435).

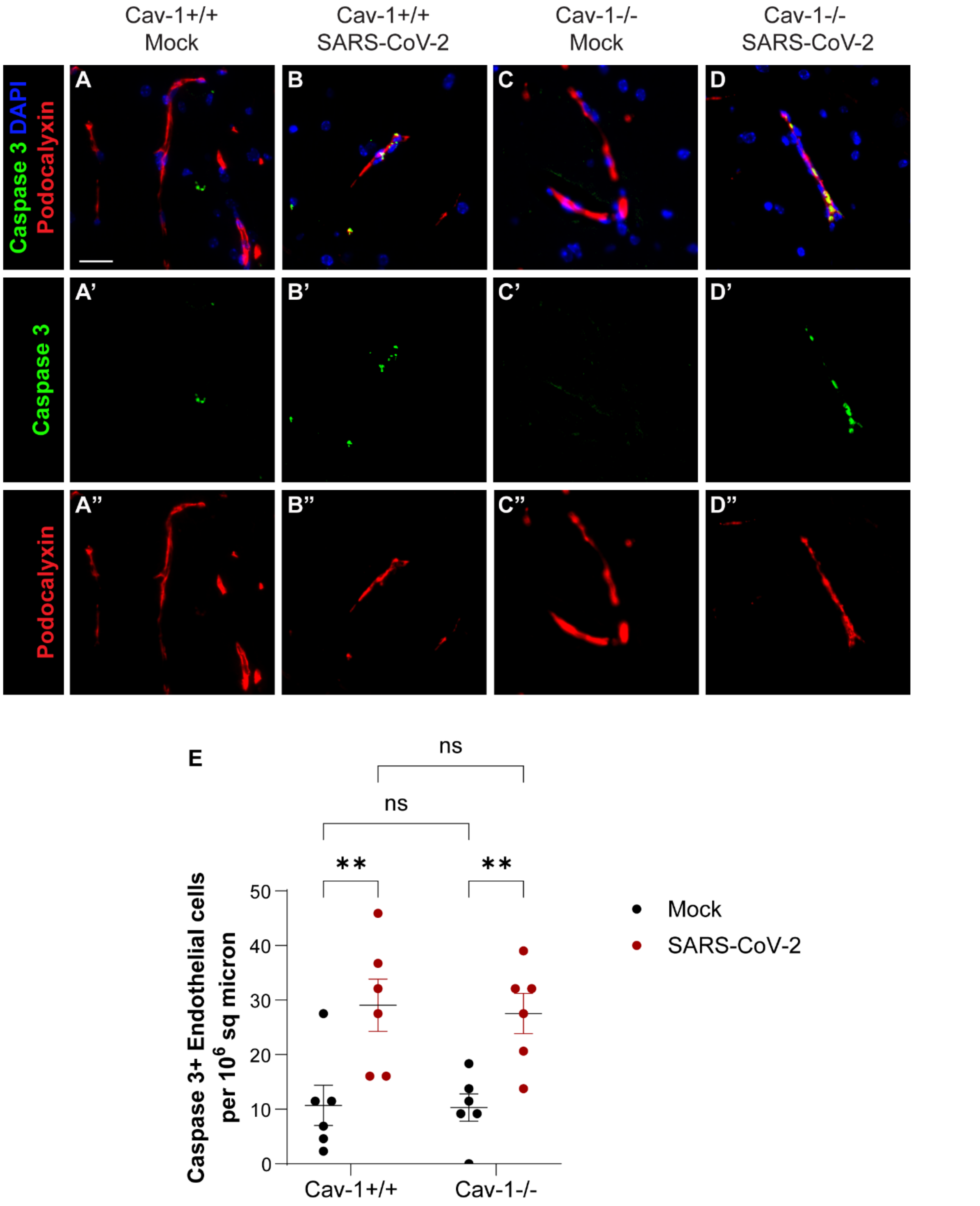

Supplementary Figure 5. Endothelial cell Caspase 3 immunoreactivity is increased in SARS-CoV-2 infection. A-D) Immunofluorescence detection of Caspase 3 (green) in

hippocampal sections from Cav-1$^{+/+}$ and Cav-1$^{-/-}$ mice euthanized 5 days after intranasal inoculation with saline (mock) or SARS-CoV-2. Immunoreactivity for podocalyxin (red) is used to identify endothelial cells and DAPI (blue) indicates nuclei. E) Quantification of the density of cells positive for caspase 3 and podocalyxin. Two way ANOVA demonstrated significant effect of infection [$F_{(1, 20)} = 22.37$, $p = 0.0001$]. Post-hoc comparisons with Fisher's LSD test revealed significant differences between mock infection and SARS-COV-2 infection for Cav-1$^{+/+}$ mice ($p < 0.01$) and for Cav-1$^{-/-}$ mice ($p < 0.01$).
