# Supplementary figures and images for: Caveolin-1 mediates blood-brain barrier permeability, neuroinflammation, and cognitive impairment in SARS-CoV-2 infection
Source: J Neuroimmunol. Author manuscript; Available in PMC 2024 Jun 28. (PMC11212674; doi:10.1016/j.jneuroim.2024.578309)

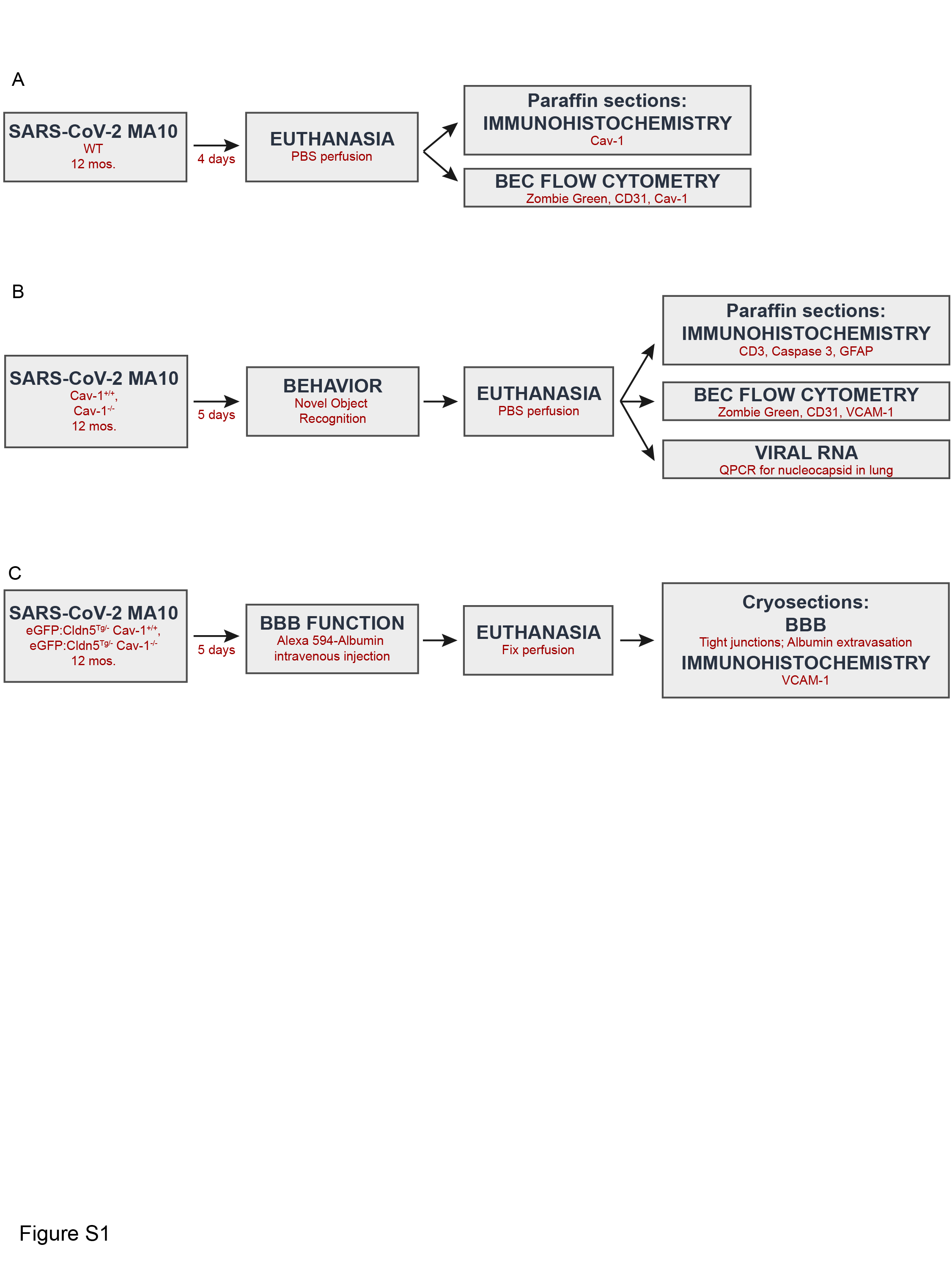

Supplement: Supplementary Figure 1. Study design. [file NIHMS1998421-supplement-Supplementary_Figure_1__Study_design_.tif]

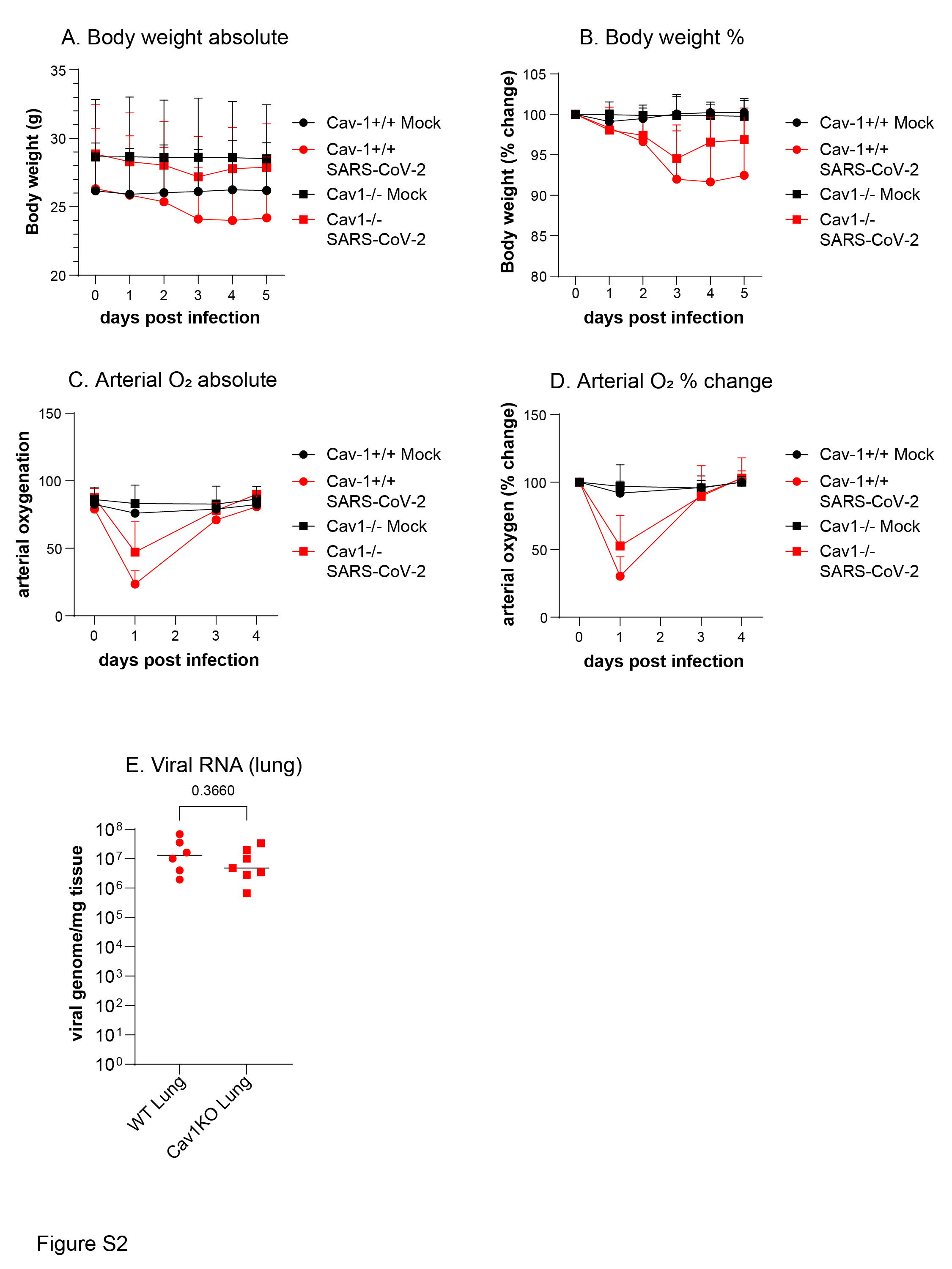

Supplement: Supplementary Figure 2. Features of SARS-CoV-2 MA10 infection. [file NIHMS1998421-supplement-Supplementary_Figure_2__Features_of_SARS-CoV-2_MA10_infection_.tif]

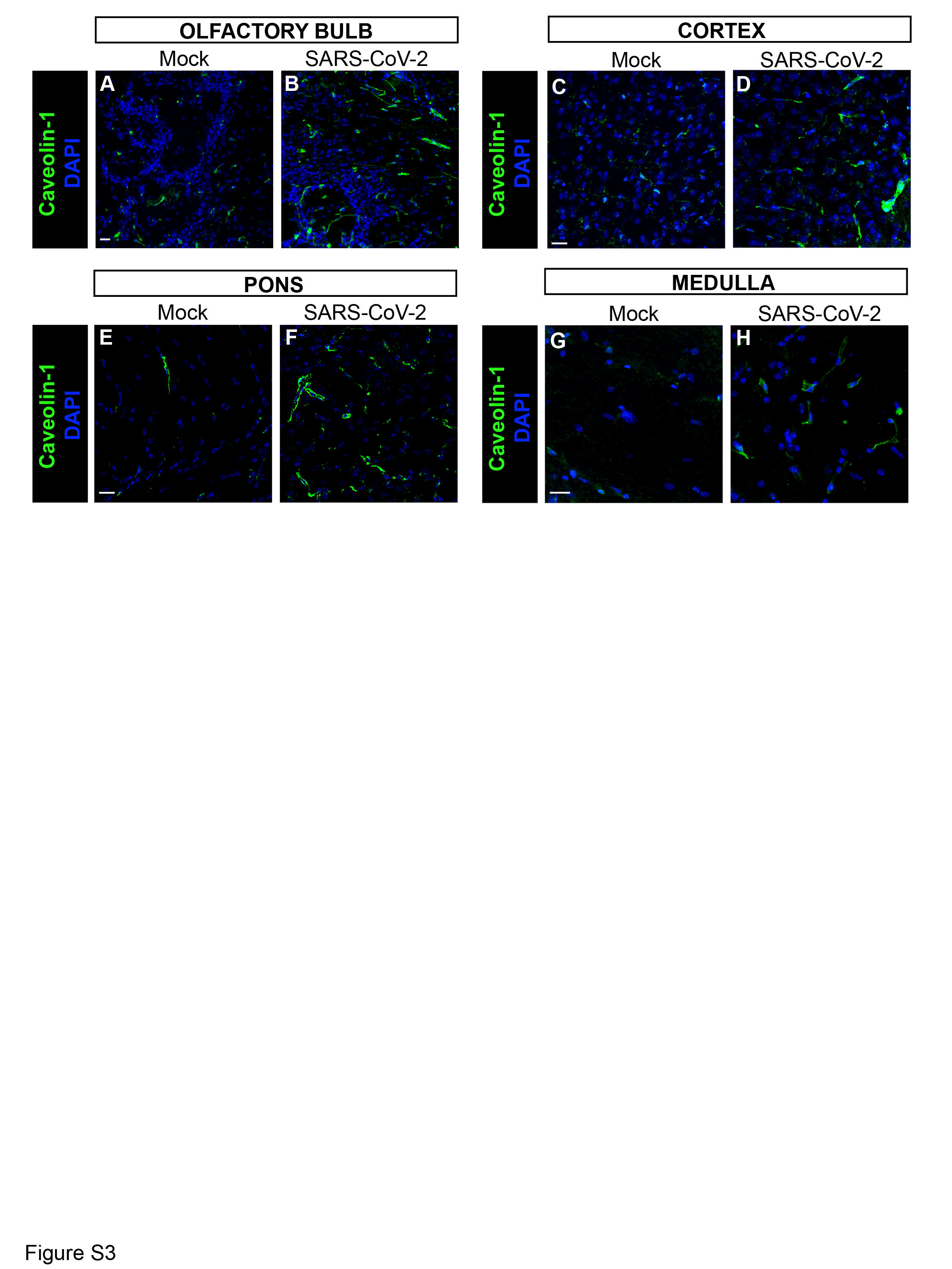

Supplement: Supplementary Figure 3. Representative micrographs of immunostaining for Cav-1. [file NIHMS1998421-supplement-Supplementary_Figure_3__Representative_micrographs_of_immunostaining_for_Cav-1_.tif]

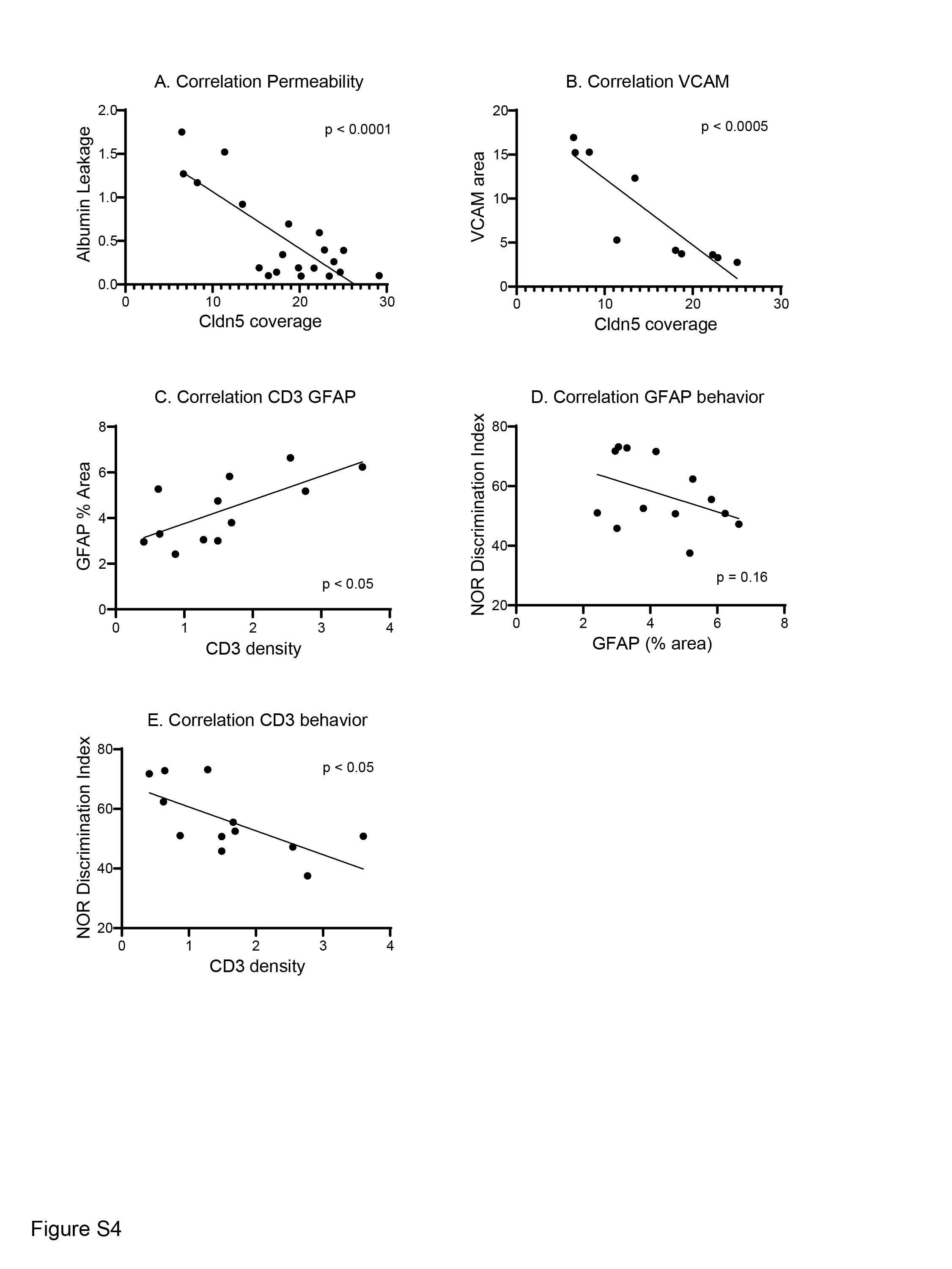

Supplement: Supplementary Figure 4. Correlations exist between features of SARS-CoV-2 infection. [file NIHMS1998421-supplement-Supplementary_Figure_4__Correlations_exist_between_features_of_SARS-CoV-2_infection_.tif]

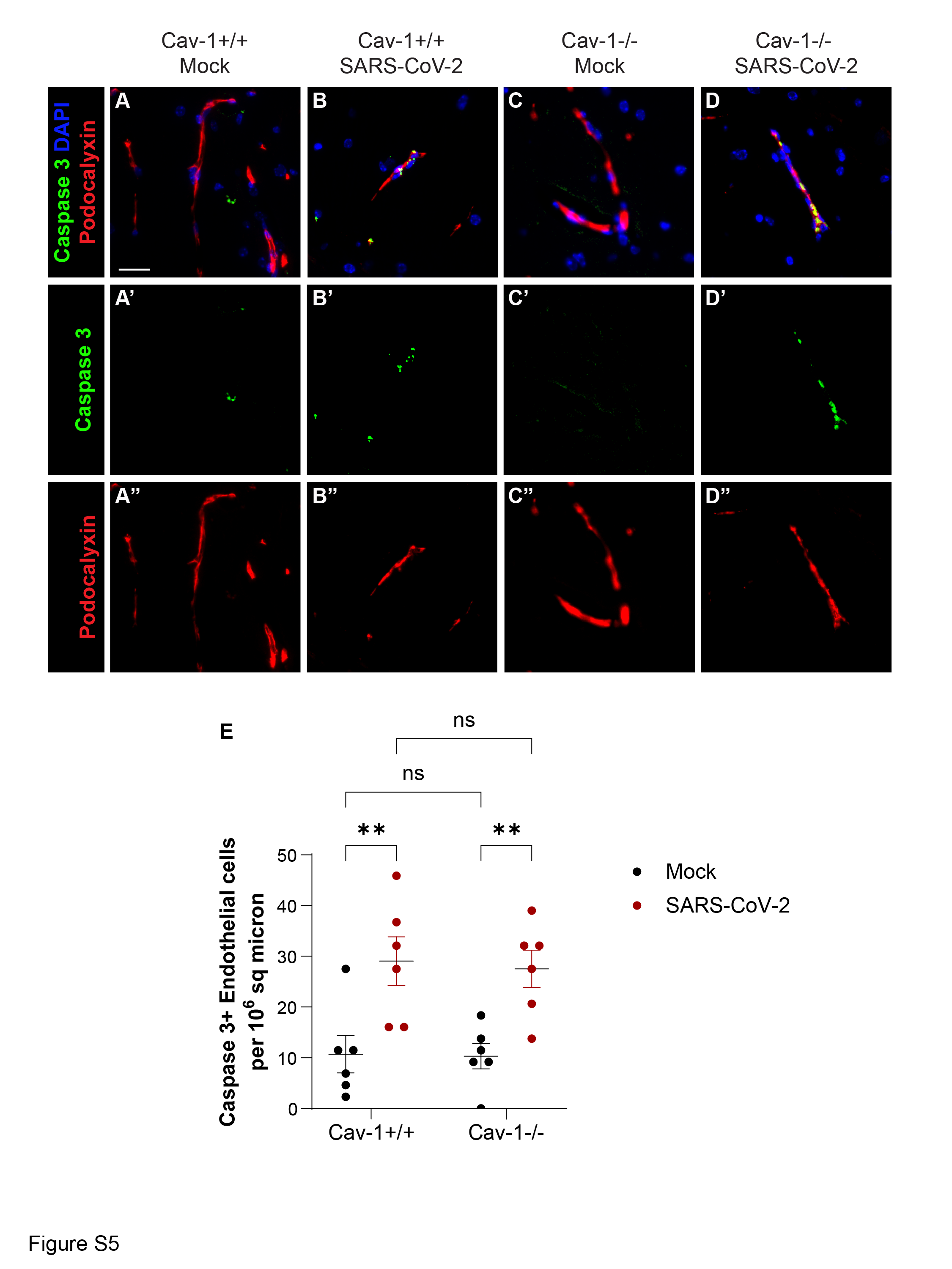

Supplement: Supplementary Figure 5. Endothelial cell Caspase 3 immunoreactivity is increased in SARS-CoV-2 infection. [file NIHMS1998421-supplement-Supplementary_Figure_5__Endothelial_cell_Caspase_3_immunoreactivity_is_increased_in_SARS-CoV-2_infection_.tif]
